# Clinical Characteristics, Racial Inequities, and Outcomes in Patients with Breast Cancer and COVID-19: A COVID-19 and Cancer Consortium (CCC19) Cohort Study

**DOI:** 10.1101/2023.03.09.23287038

**Published:** 2023-03-10

**Authors:** Gayathri Nagaraj, Shaveta Vinayak, Ali Raza Khaki, Tianyi Sun, Nicole M. Kuderer, David M. Aboulafia, Jared D. Acoba, Joy Awosika, Ziad Bakouny, Nicole B. Balmaceda, Ting Bao, Babar Bashir, Stephanie Berg, Mehmet A. Bilen, Poorva Bindal, Sibel Blau, Brianne E. Bodin, Hala T. Borno, Cecilia Castellano, Horyun Choi, John Deeken, Aakash Desai, Natasha Edwin, Lawrence E. Feldman, Daniel B. Flora, Christopher R. Friese, Matthew D. Galsky, Cyndi J. Gonzalez, Petros Grivas, Shilpa Gupta, Marcy Haynam, Hannah Heilman, Dawn L. Hershman, Clara Hwang, Chinmay Jani, Sachin R. Jhawar, Monika Joshi, Virginia Kaklamani, Elizabeth J. Klein, Natalie Knox, Vadim S. Koshkin, Amit A. Kulkarni, Daniel H. Kwon, Chris Labaki, Philip E. Lammers, Kate I. Lathrop, Mark A. Lewis, Xuanyi Li, Gilberto de Lima Lopes, Gary H. Lyman, Della F. Makower, Abdul-Hai Mansoor, Merry-Jennifer Markham, Sandeep H. Mashru, Rana R. McKay, Ian Messing, Vasil Mico, Rajani Nadkarni, Swathi Namburi, Ryan H. Nguyen, Taylor Kristian Nonato, Tracey Lynn O’Connor, Orestis A. Panagiotou, Kyu Park, Jaymin M. Patel, Kanishka GopikaBimal Patel, Jeffrey Peppercorn, Hyma Polimera, Matthew Puc, Yuan James Rao, Pedram Razavi, Sonya A. Reid, Jonathan W. Riess, Donna R. Rivera, Mark Robson, Suzanne J. Rose, Atlantis D. Russ, Lidia Schapira, Pankil K. Shah, M. Kelly Shanahan, Lauren C. Shapiro, Melissa Smits, Daniel G. Stover, Mitrianna Streckfuss, Lisa Tachiki, Michael A. Thompson, Sara M. Tolaney, Lisa B. Weissmann, Grace Wilson, Michael T. Wotman, Elizabeth M. Wulff-Burchfield, Sanjay Mishra, Benjamin French, Jeremy L. Warner, Maryam B. Lustberg, Melissa K. Accordino, Dimpy P. Shah

**Affiliations:** 1 Loma Linda University Cancer Center, Loma Linda, CA; 2 Fred Hutchinson Cancer Research Center, Seattle, WA; 3 University of Washington, Seattle, WA; 4 Seattle Cancer Care Alliance, Seattle, WA; 5 Stanford University, Palo Alto, CA; 6 Vanderbilt University Medical Center, Nashville, TN; 7 Advanced Cancer Research Group, Kirkland, WA; 8 Virginia Mason Cancer Institute, Seattle, WA; 9 University of Hawaii Cancer Center, Honolulu, HI; 10 University of Cincinnati Cancer Center, Cincinnati, OH; 11 Dana-Farber Cancer Institute, Boston, MA; 12 The University of Kansas Cancer Center, Kansas City, KS; 13 Memorial Sloan-Kettering Cancer Center, New York, NY; 14 Sidney Kimmel Cancer Center at Thomas Jefferson University, Philadelphia, PA; 15 Loyola University Medical Center, Maywood, IL; 16 Winship Cancer Institute of Emory University, Atlanta, GA; 17 Beth Israel Deaconess Medical Center, Boston, MA; 18 Northwest Medical Specialties, Tacoma, WA; 19 Herbert Irving Comprehensive Cancer Center at Columbia University, New York, NY; 20 UCSF Helen Diller Family Comprehensive Cancer Center at the University of California at San Francisco, San Francisco, CA; 21 Inova Schar Cancer Institute, Fairfax, VA; 22 Mayo Clinic, MN; 23 ThedaCare Cancer Care, Appleton, WI; 24 University of Illinois Hospital & Health Sciences System, Chicago, IL; 25 St. Elizabeth Healthcare, Edgewood, KY; 26 University of Michigan Rogel Cancer Center, Ann Arbor, MI; 27 Tisch Cancer Institute at the Icahn School of Medicine at Mount Sinai, New York, NY; 28 Cleveland Clinic, Cleveland, OH; 29 The Ohio State University Comprehensive Cancer Center, Columbus, OH; 30 Henry Ford Cancer Institute, Henry Ford Hospital, Detroit, MI; 31 Mount Auburn Hospital, Cambridge, MA; 32 Penn State Health St. Joseph Cancer Center, PA; 33 Mays Cancer Center at UT Health San Antonio MD Anderson Cancer Center, San Antonio, TX; 34 Brown University and Lifespan Cancer Institute, Providence, RI; 35 Stritch School of Medicine at Loyola University, Maywood, IL; 36 Masonic Cancer Center at the University of Minnesota, Minneapolis, MN; 37 Baptist Cancer Center, Memphis, TN; 38 Intermountain Health Care, Salt Lake City, UT; 39 Sylvester Comprehensive Cancer Center at the University of Miami Miller School of Medicine, Miami, FL; 40 Albert Einstein College of Medicine, Montefiore Medical Center, Bronx, NY; 41 Kaiser Permanente Northwest, OR/WA; 42 University of Florida, Division of Hematology and Oncology, UF Health Cancer Center, Gainesville, FL; 43 Moores Cancer Center, University of California, San Diego, CA; 44 Division of Radiation Oncology, George Washington University, Washington, DC; 45 Hartford HealthCare Cancer Institute, Hartford, CT; 46 Roswell Park Comprehensive Cancer Center, Buffalo, NY; 47 UC Davis Comprehensive Cancer Center at the University of California at Davis, CA; 48 Massachusetts General Hospital, Boston, MA; 49 Virtua Health, Marlton, NJ; 50 Division of Cancer Control and Population Sciences, National Cancer Institute, Rockville, USA; 51 Carl & Dorothy Bennett Cancer Center at Stamford Hospital, Stamford, CT; 52 METAvivor; 53 Aurora Cancer Care, Advocate Aurora Health, Milwaukee, WI; 54 Yale Cancer Center at Yale University School of Medicine, New Haven, CT

**Keywords:** Breast cancer, SARS-CoV-2, COVID-19, Risk Factors, Racial Inequities, Oncology, Pandemic

## Abstract

**Background:**

Limited information is available for patients with breast cancer (BC) and coronavirus disease 2019 (COVID-19), especially among underrepresented racial/ethnic populations.

**Methods:**

This is a COVID-19 and Cancer Consortium (CCC19) registry-based retrospective cohort study of females with active or history of BC and laboratory-confirmed severe acute respiratory syndrome coronavirus-2 (SARS-CoV-2) infection diagnosed between March 2020 and June 2021 in the US. Primary outcome was COVID-19 severity measured on a five-level ordinal scale, including none of the following complications, hospitalization, intensive care unit admission, mechanical ventilation, and all-cause mortality. Multivariable ordinal logistic regression model identified characteristics associated with COVID-19 severity.

**Results:**

1,383 female patient records with BC and COVID-19 were included in the analysis, the median age was 61 years, and median follow-up was 90 days. Multivariable analysis revealed higher odds of COVID-19 severity for older age (aOR per decade, 1.48 [95% CI, 1.32 – 1.67]); Black patients (aOR 1.74; 95 CI 1.24–2.45), Asian Americans and Pacific Islander patients (aOR 3.40; 95 CI 1.70 – 6.79) and Other (aOR 2.97; 95 CI 1.71–5.17) racial/ethnic groups; worse ECOG performance status (ECOG PS ≥2: aOR, 7.78 [95% CI, 4.83 – 12.5]); pre-existing cardiovascular (aOR, 2.26 [95% CI, 1.63 – 3.15])/pulmonary comorbidities (aOR, 1.65 [95% CI, 1.20 – 2.29]); diabetes mellitus (aOR, 2.25 [95% CI, 1.66 – 3.04]); and active and progressing cancer (aOR, 12.5 [95% CI, 6.89 – 22.6]). Hispanic ethnicity, timing and type of anti-cancer therapy modalities were not significantly associated with worse COVID-19 outcomes. The total all-cause mortality and hospitalization rate for the entire cohort was 9% and 37%, respectively however, it varied according to the BC disease status.

**Conclusions:**

Using one of the largest registries on cancer and COVID-19, we identified patient and BC related factors associated with worse COVID-19 outcomes. After adjusting for baseline characteristics, underrepresented racial/ethnic patients experienced worse outcomes compared to Non-Hispanic White patients.

The COVID-19 pandemic has had a devastating impact worldwide and within the United States (U.S.).[1], [2] Previous studies have reported that patients with cancer are at an increased risk for SARS-CoV-2 infection and have higher rates of adverse outcomes with mortality rates ranging from 14% to 33%.[3]–[10] COVID-19 has also highlighted the long-standing health inequities in the U.S, as underrepresented racial and ethnic populations have disproportionately been affected. Some studies have reported non-White race/ethnicity to be an independent risk factor for worse COVID-19 outcomes such as hospitalization and death.[3], [6], [11]–[20] Recently published data from CCC19 also showed that Black patients with cancer experienced worse COVID-19 outcomes compared to White patients after adjusting for key risk factors including cancer status and comorbidities.[21]

Breast cancer (BC) is the most common cancer diagnosed in females and affects all major racial/ethnic groups.[22]–[24] There are well described racial/ethnic differences in BC incidence and outcomes in females in the U.S attributable to multiple social and biological factors.[25]–[27] Few studies have specifically evaluated the impact of COVID-19 in patients with BC; interpretation from prior studies has been limited by small sample sizes.[28], [29] Data specifically on the impact of COVID-19 among underrepresented racial/ethnic groups with BC are also lacking. Understanding the sociodemographic and clinical factors associated with higher risk for adverse COVID-19 outcomes will help guide patient care. Hence, we aimed to evaluate the prognostic factors, racial disparities, interventions, complications, and outcomes among patients with active or previous history of BC diagnosed with COVID-19.

## METHODS

### Study population

The COVID-19 and Cancer Consortium (CCC19) consists of 129 member institutions capturing granular, detailed, and uniform data on demographic and clinical characteristics, treatment information, and outcomes of COVID-19. Details of CCC19 protocol, data collection, and quality assurance have been previously described.[30], [31] This registry-based retrospective cohort study included all female adults (age≥18 years) with an active or previous history of invasive BC and laboratory-confirmed diagnosis of SARS-CoV-2 by polymerase chain reaction (PCR) and/or serology from March 17, 2020, to June 16, 2021 in the United States. Patient records with multiple invasive malignancies including history of multiple invasive BC were excluded; patients with unknown or missing race and ethnicity, inadequate data quality (quality score>4) and those not evaluable for the primary ordinal outcome were also excluded (supplementary appendix).[31] This study was exempt from institutional review board (IRB) review (VUMC IRB#200467) and was approved by IRBs at participating sites per institutional policy. CCC19 registry is registered on ClinicalTrials.gov, NCT04354701.

### Outcome definitions

The primary outcome was a five-level ordinal scale of COVID-19 severity based on each individual patient’s most severe reported disease status: none of the following complications (0); admitted to the hospital; admitted to an intensive care unit (ICU); mechanically ventilated at any time after COVID-19 diagnosis; or death from any cause. Other COVID-19 related complications (cardiovascular; gastrointestinal; and pulmonary complications, acute kidney injury, multisystem organ failure, superimposed infection, sepsis, any bleeding); 30-day mortality; and anti-COVID-19 directed interventions (supplemental oxygen, remdesivir, systemic corticosteroids, hydroxychloroquine, and other treatments) are also reported.

### Covariates

Covariates were selected *a priori* and included: age; sex; race/ethnicity (Non-Hispanic White [NHW], Black, Hispanic, Asian Americans and Pacific Islanders [AAPI], and Other) as recorded in the EHR, based on the Center for Disease Control and Prevention Race and Ethnicity codes[32]; U.S census region of reporting institution (Northeast [NE], Midwest [MW], South and West); month/year of COVID-19 diagnosis (classified into 4-month intervals); smoking status; obesity; comorbidities (cardiovascular, pulmonary, renal, or diabetes mellitus); Eastern Cooperative Oncology Group (ECOG) performance status (PS); BC subtypes based on hormone receptor (HR) and human epidermal growth factor receptor 2 (HER2) expression (HR+/HER2-, HR+/HER2+, HR-/HER2+, HR-/HER2- [triple negative], missing/unknown); cancer status at time of COVID-19 diagnosis; timing of most recent anti-cancer therapy relative to COVID-19 diagnosis (never or after COVID-19 diagnosis, 0–4 weeks, 1–3 months, >3 months); and modality of anti-cancer therapy received within three months of COVID-19 diagnosis. Cancer status was defined as remission or no evidence of disease (NED) for >5 years, remission or NED for ≤ 5 years, and active disease, with active disease further classified as responding to therapy, stable, or progressing. Anti-cancer modalities were categorized as chemotherapy; cyclin-dependent kinase (CDK) 4/6 inhibitor; anti-HER2 therapy; other targeted therapy (non-CDK 4/6 inhibitor, non-anti-HER2 therapy); endocrine therapy; immunotherapy; and locoregional therapy (surgery and/or radiation). In the survey, drug classes (modalities) along with a few specific drugs (through checkboxes) were captured. Survey respondents were also encouraged to provide additional details in the free text boxes which were reviewed extensively by the Informatics Core at VUMC, and queries were sent to participating sites to clarify ambiguous reports. CDK 4/6 inhibitor, anti-HER2 therapy and other targeted therapy information was extracted from free text in the registry survey while the others were checkboxes. In addition, baseline severity of COVID-19 at presentation, classified as mild (no hospitalization indicated), moderate (hospitalization indicated), and severe (ICU admission indicated) was collected. Other variables included location of patient residence (urban, suburban, rural) and treatment center characteristics (academic medical center, community practice, tertiary care center). The CCC19 data dictionary is available at https://github.com/covidncancer/CCC19_dictionary. The project approved variables used for the analysis are provided in supplementary appendix.

### Statistical methods

Covariates, outcome definitions, and statistical analysis plan were pre-specified by the authors and the CCC19 Research Coordinating Center prior to analysis (supplementary appendix). Standard descriptive statistics were used to summarize prognostic factors, rates of clinical complications, interventions during hospitalization, and rates of outcomes such as 30-day mortality, hospitalization, oxygen requirement, ICU admission, mechanical ventilation, and overall mortality among racial and ethnic groups. The primary analysis was restricted to females with BC.

Multivariable ordinal logistic regression models for the COVID-19 severity outcome among females with BC included age, race/ethnicity, obesity, ECOG PS, co-morbidities, cancer status, anti-cancer therapy and timing, month/year of COVID-19 diagnosis (classified into 4-month intervals), and U.S census region of reporting institution. These covariates were identified *a priori* as the most clinically relevant for COVID-19 severity and were included in a single model, given a sufficient number of events and corresponding degrees of freedom. Because the ordinal outcome was assessed over a given patients’ total follow-up period, the model included an offset for (log) follow-up time. The results are presented as adjusted odds ratio (ORs) with 95% CIs. Model stability was assessed by comparing unadjusted and adjusted models and variance inflation factors. Graphical methods were used to verify the proportional odds assumption (supplementary appendix-figure I). We used the e value to quantify sensitivity to unmeasured confounding for the observed OR for race/ethnicity.[33], [34] Multiple imputation (20 imputed datasets) was used to impute missing and unknown data for all variables included in the analysis, with some exceptions: unknown ECOG performance score and unknown cancer status were not imputed and treated as a separate category in analyses. Imputation was performed on the largest dataset possible (that is, after removing test cases and other manual exclusions, but before applying specific exclusion criteria). Analyses were completed using R v4.0.4 (R Foundation for Statistical Computing, Vienna, Austria), including the rms and EValue extension packages. Descriptive statistics for males with BC and females with metastatic BC are presented separately but multivariable modeling was not attempted due to small sample sizes.

## RESULTS

### Baseline characteristics and COVID-19 Outcomes in female patients with BC

Of the total 12,034 reports on all cancers submitted to the CCC19 registry at the time of this analysis, 1,383 females with BC met the eligibility criteria and were included (Figure I). The median age for the cohort was 61 years (IQR 51–72 years) and median follow-up was 90 (IQR 30–135) days. BC subtypes by biomarker distribution in CCC19 registry included: 52% HR+/HER2-, 14% HR+/HER2+, 8% HR-/HER2+, 11% triple negative, and 14% unknown or missing. BC subtype distribution based on biomarkers in the CCC19 cohort are similar to SEER data which adds broader applicability of these findings.[35] With regards to BC status, 27% were in remission/NED for over 5 years and 32% were in remission/NED for less than 5 years since the initial BC diagnosis and 32% had active cancer (13% had active and responding, 12% had active and stable and 7% had active and progressing cancer). 57% of patients had received some form of anti-cancer therapy within 3 months of COVID-19 diagnosis. The unadjusted total all-cause mortality and hospitalization rate, included in the primary ordinal outcome, for the female cohort was 9% and 37%, respectively. However, the unadjusted rates of COVID-19 outcomes varied by their BC status; females with active and progressing cancer had the highest all-cause mortality (38%) and hospitalization rates (72%) compared to the rest of the group (supplementary appendix - table I*)*. Other clinical outcomes for the female cohort included 30-day all-cause mortality (6%), mechanical ventilation (5%) and ICU care (8%). Additional details on patients with BC and COVID-19 by specific characteristics of interest are presented below.

### Characteristics of female patients with BC and COVID-19 by Race/Ethnicity

Of the 1383 female patients, 736 (53%) were NHW, 289 (21%) Black, 235 (17%) Hispanic, 45 (3%) AAPI, and 78 (6%) belonged to Other racial/ethnic group. Baseline characteristics of females stratified by race/ethnicity groups are shown in Table I. Hispanic and AAPI patients were younger with median ages of 53 (IQR 46–62) and 54 (IQR 43–73) years respectively, compared to 64 years in NHW (IQR 54–76) and 61 years (IQR 52–69) in Black patients. Prevalence of smokers were higher among NHW (35%), Black (33%) and Other (32%) racial/ethnic groups compared to Hispanic (23%) and AAPI (18%) patients. Rates of obesity was higher in Black (54%) and lower in AAPI (29%) compared to NHW (42%) patients. Cardiovascular comorbidity was less common in Hispanic patients (6%), while diabetes mellitus was more prevalent among Black patients (34%) compared to NHW patients (24% and 17% respectively). Compared to NHW, Hispanic patients had higher rates of active cancer (24% responding, 15% stable, and 9% progressing) and had higher rates of receipt of anti-cancer systemic therapy within 3 months of COVID-19 diagnosis (37% chemotherapy, 25% targeted therapy, 39% endocrine therapy). Similarly, AAPI patients also had higher rates of active cancer (7% responding, 22% stable, and 13% progressing) and received anti-cancer systemic therapy within 3 months of COVID-19 diagnosis (24% chemotherapy, 18% targeted therapy, 33% endocrine therapy) compared to NHW patients with active cancer [9% responding, 12% stable, and 6% progressing] who received anti-cancer systemic therapy [16% chemotherapy, 15% targeted therapy, 38% endocrine therapy]). With regards to baseline severity of COVID-19 at presentation, 39% of Black and 38% of AAPI patients presented with moderate or higher severity of COVID-19 infection compared to 27% in both NHW and Hispanic patients. Table II summarizes the clinical outcomes, complications, and interventions, stratified by race/ethnicity.

### Characteristics of female patients with Metastatic Breast Cancer (MBC) and COVID-19

Female patients with MBC consisted of 17% of the cohort (N=233), with median age 58 years [IQR 50–68]. Racial/Ethnic groups consisted of 46% NHW, 24% Black, 21% Hispanics, 4% AAPI and 4% Other. Most patients with MBC were never smokers (70%) and non-obese (60%). The predominant tumor biology was HR+/HER2- (42%) followed by HR+/HER2+(23%). The most common sites of metastases were bone (58%), lung (28%), and liver (26%). A high percentage (87%) had received anti-cancer treatment within 3 months prior to COVID-19 diagnosis and 32% had active and progressing cancer. The unadjusted total all-cause mortality and hospitalization rate in females with MBC was 19% and 53% respectively. Further details of baseline characteristics and unadjusted rates of COVID-19 outcomes, complications and interventions are presented in supplementary appendix – Table IIA and IIB

### BC Treatment Characteristics

758 (55%) out of 1383 female patients with BC received some form of systemic treatment within 3 months prior to COVID-19 diagnosis, and specific drug information was available for 679 (90%) (Table III). Of these 679 patients, the most common systemic therapy was endocrine therapy alone (n=336, 49.5%). This was followed by chemotherapy in 163 (24%) patients who received it either as single agent (n=55, 8%) or combination chemotherapy (n=60, 9%) or combined with anti-HER2 therapy (n=48, 7%). 78 (11.5%) patients received anti-HER2 therapy with or without endocrine therapy, and 63 (9%) patients received CDK4/6 inhibitors with or without endocrine therapy.

### Prognostic Factors associated with COVID-19 severity

After adjusting for baseline demographic, clinical, and spatiotemporal factors in multivariable analysis model, factors associated with worse outcomes in females with BC included older age (aOR per decade, 1.48 [95% CI, 1.32 – 1.67]); Black (aOR, 1.74 [95% CI, 1.24 – 2.45]), AAPI (aOR, 3.40 [95% CI, 1.70 – 6.79]), and Other (aOR, 2.97 [95% CI, 1.71 – 5.17]) racial/ethnic group; cardiovascular (aOR, 2.26 [95% CI, 1.63 – 3.15]) and pulmonary (aOR, 1.65 [95% CI, 1.20 – 2.29]) comorbidities; diabetes mellitus (aOR, 2.25 [95% CI, 1.66 – 3.04]); worse ECOG PS (ECOG PS 1: aOR, 1.74 [95% CI, 1.22 – 2.48]; ECOG PS ≥2: aOR, 7.78 [95% CI, 4.83 – 12.5]); and active and progressing cancer status (aOR, 12.5 [95% CI, 6.89 – 22.6]). Association between Hispanic ethnicity, obesity, preexisting renal disease, anti-cancer treatment modalities including all forms of systemic therapy and loco-regional therapy, month/year and geographic region of COVID-19 diagnosis and COVID-19 severity did not reach statistical significance (Table IV). The e value for the COVID-19 severity OR and CI for each racial group are shown in supplementary appendix - Table III. This value demonstrates the impact of unknown _*residual*_ confounding above that adjusted for by including adjustment variables in the multivariable model. For example, an unmeasured confounder would need to be associated with both race and mortality with a odds ratio of at least 1.97 to fully attenuate the observed association for Black females and the odds ratio would need to be at least 1.47 for the null-hypothesized value (1.0) to be included in the CI. Similarly, E value estimates are noted for AAPI and Other groups. The unmeasured confounding for other races based on the e value is larger than most documented associations in the CCC19 cohort. [3]

### Male patients with BC and COVID-19

Male patients with BC were evaluated separately as part of exploratory analysis. The median age for male BC cohort (N=25) was 67 years [IQR 60–75]. Racial/ethnic composition consisted of NHW (52%) followed by Black (32%) males. Most males with BC were non-smokers (72%) and diabetes mellitus was the predominant comorbidity (44%). The hospitalization rate was 60% and all-cause mortality was 20%. Additional clinical characteristics, complications, interventions, and unadjusted outcomes among males with BC in the CCC19 registry are provided in supplementary appendix – table IV.

## DISCUSSION

In this large, multi-institutional and racially diverse cohort of females with BC and COVID-19 from CCC19 registry, we assessed the clinical impact of COVID-19. The all-cause mortality from COVID-19 was 9% and hospitalization rate was 37%, which is numerically lower than in the entire CCC19 cohort at 14% and 58%, and other previously reported studies of COVID-19 in patients with cancer.[3]–[8], [36] These differences in outcomes could indicate differences in the immunocompromised status of patients due to intensity of therapy regimens, complex comorbidities, or concomitant medications, which may affect outcomes. Females with BC, however, form a heterogenous group, and the rates of outcomes varied widely with their disease status; patients with active and progressing cancer had the highest total all-cause mortality (38%) and hospitalization rates (72%).

We observed older age, pre-existing cardiovascular and pulmonary comorbidities, diabetes mellitus, worse ECOG PS, and active and progressing cancer status were associated with adverse COVID-19 outcomes in females with BC. Prior studies have reported similar factors to be associated with adverse COVID-19 outcomes in patients with all cancer types. The majority of these studies have reported older age to be an important prognostic factor for adverse outcomes from COVID-19, including mortality, which is consistent with data presented here.[3], [9], [10], [36], [37] Non-cancer comorbidities, contributing to poor COVID-19 outcomes, as noted in our study, have also been a consistent finding in patients with and without a cancer diagnosis.[3], [9], [10], [37], [38] Similarly, poor ECOG PS in cancer patients has been noted to be an important factor associated with worse COVID-19 severity, including our study.[3], [8], [10] While obesity was reported in some cancer studies to have a negative impact on COVID-19 [3], [37], our study did not identify this association. In this cohort of females with BC, all forms of anti-cancer therapy were thoroughly evaluated and none of the systemic therapies including chemotherapy; endocrine therapy; and targeted therapy (anti-HER2, CDK4/6 inhibitors, other non-HER2 or non-CDK4/6 inhibitors); or loco-regional therapy (surgery and radiation) received within 3 months of COVID-19 diagnosis was significantly associated with adverse COVID-19 outcomes. Our finding suggests that systemic therapy for females with BC may not add excess COVID-19 risk. Multiple large cohort studies and meta-analysis of patients with cancer diagnosed with COVID-19 similarly did not identify active anti-cancer therapy, specifically chemotherapy, as a factor associated with adverse COVID-19 outcomes, which is consistent with our results.[4], [5], [8], [36], [39], [40] However, in contrast, some studies of patients with other cancers have shown a negative impact of chemotherapy[3], [9], [10], [37] and immunotherapy use [37]. These findings have important clinical implications while counselling and providing patient care during the pandemic.

We also report important findings related to the impact of racial/ethnic inequities in females with BC and COVID-19, which adds to the growing body of literature on COVID-19 related racial/ethnic disparities. In our study, Black females with BC had significantly worse COVID-19 outcomes compared to NHW females. Multiple studies have similarly reported Black patients in US with and without cancer diagnosis having significantly worse COVID-19 outcomes [3], [6], [11] however, our study is the first to show such racial/ethnic disparities in COVID-19 outcomes in females with BC. There was no statistically significant association of worse outcomes for Hispanic females compared to NHW females. This is different in comparison to our overall CCC19 cohort[3], and may be explained by younger age and lower rates of comorbid conditions in Hispanic females compared to NHW females. We also found females belonging to AAPI, and Other racial/ethnic group to have worse COVID-19 outcomes. Notably, females belonging to Black, AAPI and Other racial/ethnic groups presented with higher rates of moderate or severe symptoms of COVID-19 at baseline, which likely contributed to their worse outcomes. This in turn is possibly related to barriers to health care access, and other socio-cultural reasons for delay in seeking early medical care. Future studies including social determinants of health, access to health care, and lifestyle behaviors, among others, are warranted to identify barriers contributing to worse clinical presentation in racial/ethnic minority groups, and eventually impacting future health policies.

In summary, this is one of the largest cohort studies to evaluate the clinical impact of COVID-19 on females with BC. Strengths of our study include standardized data collection on the most common cancer in females in the US and large sample size to evaluate the effect of major clinical and demographic factors. The study had representative population by race and ethnicity from geographically diverse areas and variable time/period of COVID-19 diagnosis. In addition, our study has detailed manually collected information on both cancer status and treatment modalities which contrasts with other studies that have utilized either of these variables as surrogate. Limitations of this study include the retrospective nature of data and inherent potential for confounding because of its observational nature. It’s possible that ascertainment bias could have led to some of the high values observed in specific groups such as females with MBC and those with active and progressing cancer. Additional information on drivers for inequity such as socio-economic status, occupation, income, residence, education, and insurance status may have provided added insights on the root causes for disparities; however, unavailability of these factors does not nullify our current findings of existing racial disparities in COVID-19 outcomes in females with BC. Vaccination status was not part of this study as vaccines were not available during the predominant time frame for this cohort. Data presented here including the risk of hospitalization and death applies to the specific COVID-19 variants prevalent during the study period. Despite these limitations, the study reports important sociodemographic and clinical factors that aid in identifying females with BC who are at increased risk for severe COVID-19 outcomes.

Our study addresses an important knowledge gap in patients with BC diagnosed with COVID-19 using the CCC19 registry. In addition to clinical and demographic factors associated with adverse COVID-19 outcomes, racial/ethnic disparities reported here significantly contribute to the growing literature. At this stage, it is irrefutable that one of the principal far-reaching messages the pandemic has conveyed is that any such major stressors on the health care system increases risk of detrimental outcomes to the most vulnerable patient population, including the underrepresented and the underserved. These are important considerations for future resource allocation strategies and policy interventions. We also report an important finding that cancers that are active and progressing are associated with severe COVID-19 outcomes. During the ongoing pandemic, this has significant implications for shared decision-making between patients and physicians.

## Supplementary Material

Supplement 1

## Figures and Tables

**Table I:** Baseline Characteristics by Race/Ethnicity

	NHW	Black	Hispanic	AAPI	Others	All
	(n = 736, 53%)	(n = 289, 21%)	(n = 235, 17%)	(n = 45, 3%)	(n=78, 6%)	(n = 1383, 100%)
**Median Age, years**^a^ **[IQR]**	64 (54–76)	61 (52–69)	53 (46–62)	54 (43–73)	62 (53–71)	61 (51–72)
**Median follow-up, days [IQR]**	90 (30–135)	90 (30–180)	90 (30–135)	42 (21–90)	70 (30–180)	90 (30–135)
**Smoking status**						
Never	460 (62%)	186 (64%)	180 (77%)	35 (78%)	50 (64%)	911 (66%)
Current or Former	261 (35%)	95 (33%)	53 (23%)	8 (18%)	25 (32%)	442 (32%)
Missing/unknown	15 (2%)	8 (3%)	2 (1%)	2 (4%)	3(4%)	30 (2%)
**Obesity**						
No	421 (57%)	133 (46%)	116 (49%)	32 (71%)	45 (58%)	747 (54%)
Yes	308 (42%)	156 (54%)	116 (49%)	13 (29%)	33 (42%)	626 (45%)
Missing/unknown	7 (1%)	0 (0%)	3 (1%)	0 (0%)	0 (0%)	10 (1%)
**Comorbidities** ^b^						
Cardiovascular	179 (24%)	60 (21%)	14 (6%)	7 (16%)	11 (14%)	271 (20%)
Pulmonary	125 (17%)	65 (22%)	33 (14%)	<5 (<11%)	7 (9%)	234 (17%)
Renal Disease	66 (9%)	31 (11%)	13 (6%)	<5 (<11%)	<5 (<6%)	115 (8%)
Diabetes mellitus	127 (17%)	98 (34%)	51 (22%)	10 (22%)	20 (26%)	306 (22%)
Missing/unknown	9 (1%)	1 (<1%)	5 (2%)	0 (0%)	0 (0%)	15 (1%)
**ECOG performance status**						
0	314 (43%)	130 (45%)	123 (52%)	18 (40%)	32 (41%)	617 (45%)
1	135 (18%)	72 (25%)	48 (20%)	10 (22%)	16 (21%)	281 (20%)
2+	69 (9%)	33 (11%)	15 (6%)	5 (11%)	5 (6%)	127 (9%)
Unknown	218 (30%)	53 (18%)	49 (21%)	12 (27%)	25 (32%)	357 (26%)
Missing	0 (0%)	1 (<1%)	0 (0%)	0 (0%)	0 (0%)	1 (<1%)
**Region**						
Northeast	247 (34%)	101 (35%)	106 (45%)	12 (27%)	26 (33%)	492 (36%)
Midwest	239 (32%)	110 (38%)	23 (10%)	8 (18%)	12 (15%)	392 (28%)
South	116 (16%)	58 (20%)	27 (11%)	X*	14 (18%)	218 (16%)
West	128 (17%)	16 (6%)	77 (33%)	22 (49%)	24 (31%)	267 (19%)
Undesignated	6 (1%)	4 (1%)	2 (1%)	3 (7%)*	2 (3%)	14 (1%)
**Month/Year of COVID-19 diagnosis**						
Jan-Apr 2020	140 (19%)	74 (26%)	41 (17%)	8 (18%)	20 (26%)	283 (20%)
May-Aug 2020	279 (38%)	141 (49%)	101 (43%)	24 (53%)	30 (38%)	575 (42%)
Sept-Dec 2020	197 (27%)	42 (15%)	50 (21%)	5 (11%)	16 (21%)	310 (22%)
Jan-Jun 2021	118 (16%)	32 (11%)	41 (17%)	7 (16%)	12 (15%)	210 (15%)
Missing/unknown	2 (<1%)	0 (0%)	2 (1%)	1 (2%)	0 (0%)	5 (<1%)
**Area of patient residence**						
Urban	193 (26%)	136 (47%)	124 (53%)	13 (29%)	30 (38%)	496 (36%)
Suburban	315 (43%)	77 (27%)	65 (28%)	17 (38%)	31 (40%)	505 (37%)
Rural	81 (11%)	7 (2%)	9 (4%)	X*	0 (0%)	98 (7%)
Missing/unknown	147 (20%)	69 (24%)	37 (16%)	15 (33%)*	17 (22%)	284 (21%)
**Treatment center characteristics**						
Academic Medical Center	123 (17%)	102 (35%)	43 (18%)	7 (16%)	11 (14%)	286 (21%)
Community Practice	238 (32%)	51 (18%)	44 (19%)	X*	23 (29%)	359 (26%)
Tertiary Care Center	375 (51%)	136 (47%)	147 (63%)	35 (78%)	44 (56%)	737 (53%)
Missing/unknown	0 (0%)	0 (0%)	1 (<1%)	3 (7%)*	0 (0%)	1 (<1%)
**Receptor status**						
HR+/HER2-	419 (57%)	135 (47%)	102 (43%)	22 (49%)	43 (55%)	721 (52%)
HR+/HER2+	102 (14%)	35 (12%)	43 (18%)	7 (16%)	9 (12%)	196 (14%)
HR-/HER2+	46 (6%)	28 (10%)	32 (14%)	X*	X*	111 (8%)
Triple Negative	57 (8%)	54 (19%)	35 (15%)	5 (11%)	7 (9%)	158 (11%)
Missing/unknown	112 (15%)	37 (13%)	23 (10%)	11 (24%)*	19 (24%)*	197 (14%)
**Cancer status**						
Remission/NED, >5 yrs	247 (34%)	76 (26%)	23 (10%)	9 (20%)	20 (26%)	375 (27%)
Remission/NED, <5 yrs	234 (32%)	100 (35%)	77 (33%)	11 (24%)	26 (33%)	448 (32%)
Active and responding	68 (9%)	35 (12%)	56 (24%)	X*	11 (14%)	173 (13%)
Active and stable	91 (12%)	28 (10%)	35 (15%)	10 (22%)	5 (6%)	169 (12%)
Active and progressing	41 (6%)	27 (9%)	20 (9%)	6 (13%)	X*	97 (7%)
Unknown	48 (7%)	19 (7%)	22 (9%)	6 (13%)*	15 (19%)*	104 (8%)
Missing	7 (1%)	4 (1%)	2 (1%)	3 (7%)	1 (1%)	17 (1%)
**Timing of anti-cancer therapy**						
Never/After COVID-19	24 (3%)	10 (3%)	7 (3%)	X*	7 (9%)	50 (4%)
0–4 weeks	364 (49%)	135 (47%)	158 (67%)	25 (56%)	39 (50%)	721 (52%)
1–3 months	26 (4%)	20 (7%)	19 (8%)	0 (0%)	X*	69 (5%)
>3 months	303 (41%)	118 (41%)	45 (19%)	18 (40%)	24 (31%)	508 (37%)
Missing/unknown	19 (3%)	6 (2%)	6 (3%)	2 (4%)*	8 (10%)*	35 (3%)
**Modality of active anti-cancer therapy** ^b,c^						
None	333 (45%)	127 (44%)	53 (23%)	20 (44%)	30 (38%)	563 (41%)
Chemotherapy	117 (16%)	68 (24%)	88 (37%)	11 (24%)	14 (18%)	298 (22%)
Targeted Therapy	112 (15%)	38 (13%)	59 (25%)	8 (18%)	11 (14%)	228 (16%)
*Anti-HER2 therapy*	60 (8%)	17 (6%)	36 (15%)	<5 (<11%)	<5 (<6%)	123 (9%)
*CDK4/6 inhibitor*	33 (4%)	12 (4%)	14 (6%)	<5 (<11%)	<5 (<6%)	65 (5%)
*Other* ^d^	14 (2%)	5 (2%)	<5 (<2%)	<5 (<11%)	0 (0%)	24 (2%)
Endocrine Therapy	283 (38%)	86 (30%)	91 (39%)	15 (33%)	26 (33%)	501 (36%)
Immunotherapy	12 (2%)	8 (3%)	<5 (<2%)	<5 (<11%)	<5 (<6%)	28 (2%)
Local (Surgery/Radiation)	80 (11%)	37 (13%)	41 (17%)	<5 (<11%)	9 (12%)	172 (12%)
Other	13 (2%)	3 (1%)	2 (1%)	0 (0%)	0 (0%)	18 (1%)
Missing/unknown	12 (2%)	7 (2%)	5 (2%)	0 (0%)	5 (6%)	29 (2%)
**Severity of COVID-19**						
Mild	535 (73%)	177 (61%)	173 (74%)	28 (62%)	50 (64%)	963 (70%)
Moderate	174 (24%)	97 (34%)	56 (24%)	14 (31%)	21 (27%)	362 (26%)
Severe	25 (3%)	15 (5%)	6 (3%)	X*	7 (9%)	56 (4%)
Missing/unknown	2 (<1%)	0 (0%)	0 (0%)	3 (7%)*	0 (0%)	2 (<1%)

* Cells combined to mask N<5 according to CCC19 low count policy

a Age was truncated at 90

b Percentages could sum to >100% because categories are not mutually exclusive

c Within 3 months of COVID-19 diagnosis.

d Therapies other than Anti-Her2 therapy or CDK4/6 inhibitor

Variable categories with one to five cases are masked by replacing with N < 5 according to CCC19 policy

**Table II:** Outcomes, Clinical Complications, and COVID-19 Interventions

	NHW n^a^ (%)	Black n^a^ (%)	Hispanic n^a^ (%)	AAPI n^a^ (%)	Other n^a^ (%)	All n^a^ (%)
**Outcomes**						
Total all-cause mortality^b^	60 (8)	38 (13)	12 (5)	<5(<11)	9 (12)	123 (9)
30-day all-cause mortality^c^	40 (5)	29 (10)	8 (3)	<5 (<11)	8 (10)	89 (6)
Received mechanical ventilation^b^	24 (3)	26 (9)	11 (5)	<5 (<11)	<5 (<6)	69 (5)
Admitted to an intensive care unit^b^	45 (6)	31 (11)	18 (8)	7 (16)	10 (13)	111 (8)
Admitted to the hospital^b^	245 (33)	137 (47)	77 (33)	20 (44)	33 (42)	512 (37)
**Clinical complications**						
Any cardiovascular complication^d^	82 (11)	50 (17)	30 (13)	6 (13)	18 (23)	186 (14)
Any pulmonary complication^e^	170 (23)	88 (31)	43 (18)	12 (27)	23 (30)	336 (24)
Any gastrointestinal complication^f^	12 (2)	7 (2)	<5 (<2)	<5 (<11)	<5 (<7)	26 (2)
Acute kidney injury	41 (6)	46 (16)	11 (5)	5 (11)	10 (13)	113 (8)
Multisystem organ failure	10 (1)	12 (4)	<5 (<2)	<5 (<11)	<5 (<7)	29 (2)
Superimposed infection	62 (9)	42 (15)	14 (6)	7 (16)	<5 (<7)	129 (10)
Sepsis	43 (6)	24 (8)	15 (6)	7 (16)	12 (16)	101 (7)
Any bleeding	15 (2)	7 (2)	<5 (<2)	<5 (<11)	<5 (<7)	29 (2)
**Interventions**						
Remdesivir	68 (10)	20 (7)	15 (7)	8 (18)	5 (7)	116 (9)
Hydroxychloroquine	60 (9)	41 (15)	14 (6)	<5 (<11)	11 (15)	129 (10)
Systemic Corticosteroids	107 (15)	50 (18)	31 (14)	8 (18)	13 (18)	209 (16)
Other	112 (16)	53 (19)	36 (16)	11 (25)	12 (17)	224 (17)
Supplemental oxygen	173 (24)	87 (31)	43 (19)	14 (31)	24 (31)	341 (25)

Variable categories with one to five cases are masked by replacing with N < 5 according to CCC19 policy

a N based on number of patients with non-missing data.

b Included in primary outcome

c Secondary outcome

d Cardiovascular complication includes hypotension, myocardial infarction, other cardiac ischemia, atrial fibrillation, ventricular fibrillation, other cardiac arrhythmia, cardiomyopathy, congestive heart failure, pulmonary embolism (PE), deep vein thrombosis (DVT), stroke, thrombosis NOS complication.

e Pulmonary complication includes respiratory failure, pneumonitis, pneumonia, acute respiratory distress syndrome (ARDS), PE, pleural effusion, empyema.

f Gastrointestinal complication includes acute hepatic injury, ascites, bowel obstruction, bowel perforation, ileus, peritonitis

**Table III:** Systemic Treatments Received within 3 months prior to COVID-19 Diagnosis

	N (%)
Total	679 (100%)
Endocrine therapy alone	336 (49.5)
CDK4/6 inhibitor +/− Endocrine therapy	63 (9)
Other targeted therapy +/− Endocrine therapy	10 (1.5)
Anti-HER2 therapy +/− Endocrine therapy	78 (11.5)
Anti-HER2 therapy + Chemotherapy	48 (7)
Single agent chemotherapy +/− Endocrine therapy	55 (8)
Combination chemotherapy +/− Endocrine therapy	60 (9)
Immunotherapy +/− Chemotherapy	19 (3)
Other combination therapies	10 (1.5)

**Table IV:** Adjusted Associations of Baseline Characteristics with COVID-19 Severity Outcome.

	COVID-19 severity
	OR (95% CI)
**Age (per decade)**	**1.48 (1.32– 1.67)**
**Race (Ref: Non-Hispanic White)** ^a^	
Non-Hispanic Black	**1.74 (1.24– 2.45)**
Hispanic	1.38 (0.93– 2.05)
Non-Hispanic AAPI	**3.40 (1.70– 6.79)**
Other	**2.97 (1.71– 5.17)**
**Obesity (Ref: No)**	1.20 (0.92– 1.57)
**Cardiovascular Comorbidity (Ref: No)**	**2.26 (1.63– 3.15)**
**Pulmonary Comorbidity (Ref: No)**	**1.65 (1.20– 2.29)**
**Renal Disease (Ref: No)**	1.34 (0.86– 2.07)
**Diabetes Mellitus (Ref: No)**	**2.25 (1.66– 3.04)**
**ECOG Performance Status (Ref: 0)**	
1	**1.74 (1.22– 2.48)**
2+	**7.78 (4.83–12.5)**
Unknown	**2.26 (1.61– 3.19)**
**Cancer Status (Ref: Remission/NED, >5 years)**	
Remission or NED, <5 years	0.91 (0.63– 1.33)
Active and responding	1.07 (0.63– 1.83)
Active and stable	1.37 (0.82– 2.28)
Active and progressing	**12.5 (6.89–22.6)**
Unknown	1.79 (0.96– 3.34)
**Chemotherapy (Ref: No)**	1.37 (0.91– 2.06)
**Anti-HER2 Therapy (Ref: No)**	1.13 (0.67– 1.92)
**CDK 4/6 inhibitor (Ref: No)**	1.21 (0.60– 2.42)
**Other Targeted Therapies**^b^ **(Ref: No)**	1.78 (0.69– 4.59)
**Endocrine Therapy (Ref: No)**	1.00 (0.73– 1.37)
**Locoregional Therapy (Ref: No)**	1.36 (0.88– 2.10)
**Never received cancer treatment (Ref: >3 month)**	0.65 (0.28– 1.49)
**Month/Year of COVID-19 Diagnosis (Ref: Jan-Apr 2020)**	
May-Aug 2020	0.57 (0.41– 0.81)
Sept-Dec 2020	0.45 (0.30– 0.68)
Jan-Jun 2021	0.57 (0.36– 0.89)
**Region (Ref: Northeast)**	
Midwest	0.76 (0.54– 1.05)
South	0.76 (0.51– 1.13)
West	0.43 (0.29– 0.65)

a Odds ratios greater than 1 indicate higher odds of composite outcome. The P value for evaluating the null hypothesis of equality in odds ratios across race (4 degrees of freedom) was <0.001.

b Therapies other than CDK4/6 inhibitor or Anti- HER2 therapy All variance inflation factors are <1.8 for the model

## ABBREVIATIONS:

CCC19: Cancer and COVID-19 Consortium
COVID-19: Coronavirus disease 2019
BC: Breast Cancer
SARS-CoV-2: Severe Acute Respiratory Syndrome Coronavirus-2
NHW: Non-Hispanic White
AAPI: Asian Americans and Pacific Islanders
ECOG PS: Eastern Cooperative Oncology Group performance status
HR: Hormone receptor
HER2: Human epidermal growth factor receptor 2
CDK 4/6: inhibitor Cyclin-dependent kinase 4/6 inhibitor
NED: No evidence of disease
MBC: Metastatic breast cancer
