# Supplementary material for: Clinical Characteristics, Racial Inequities, and Outcomes in Patients with Breast Cancer and COVID-19: A COVID-19 and Cancer Consortium (CCC19) Cohort Study
Source: medRxiv. 2023 Mar 10:2023.03.09.23287038. Preprint. [Version 1] doi: 10.1101/2023.03.09.23287038 (PMC10187350; doi:10.1101/2023.03.09.23287038)
Supplement: Supplement 1 [file NIHPP2023.03.09.23287038v1-supplement-1.pdf]

medRxiv preprint doi: https://doi.org/10.1101/2023.03.09.23287038; this version posted March 10, 2023. The copyright holder for this preprint (which was not certified by peer review) is the author/funder, who has granted medRxiv a license to display the preprint in perpetuity.
It is made available under a CC-BY 4.0 International license .

<u>Supplementary Appendix</u>

TABLE OF CONTENTS

APPENDIX A: CCC-19 Quality Scores **Page: 6**

APPENDIX B: Breast Cancer Disparities Statistical Analysis Plan: **Pages: 7-13**

APPENDIX C: CCC19 Approved Project Variables **Pages:14-20**

APPENDIX D: Supplementary Figure I - Proportional Odds Assumption **Page:21**

APPENDIX E: Suppl Table I – Unadjusted rates of outcomes after COVID-19 diagnosis by cancer status **Page:22**

APPENDIX F: Suppl Table IIA and IIB: Baseline Characteristics of Females with MBC and unadjusted rates of outcomes after COVID-19 diagnosis **Pages: 23-26**

APPENDIX G: Suppl Table III: Sensitivity Analysis by Race/Ethnicity **Page: 27**

APPENDIX H: Suppl Table IVA & IVB - Male Breast Cancer **Pages: 28-30**

APPENDIX I: List of Participants by Institution **Pages: 31-34**

medRxiv preprint doi: https://doi.org/10.1101/2023.03.09.23287038; this version posted March 10, 2023. The copyright holder for this preprint (which was not certified by peer review) is the author/funder, who has granted medRxiv a license to display the preprint in perpetuity.
It is made available under a CC-BY 4.0 International license .

APPENDIX A: CCC-19 Quality Scores

The CCC-19 uses a quality scoring system to determine the suitability of records for inclusion in analyses. A score greater than 5 was considered insufficient for inclusion in the analysis presented. Scores are tabulated as follows:

Minor problems (+1 points per problem)
ADT missing/unknown (prostate cancers only)
Biomarkers missing/unknown (breast cancers only)
ICU admission missing/unknown
Hospitalization missing/unknown
Mechanical ventilation missing/unknown
O2 ever needed missing/unknown
Days to death missing/unknown
Cancer status unknown
ECOG PS unknown
Missing cancer drug names for patients on systemic anti-cancer treatment
Missing or unknown categorical lab values if labs were drawn

Moderate problems (+3 points per problem)
Cancer status missing
ECOG PS missing
Death status missing/unknown
Baseline COVID-19 severity missing/unknown
Should have 30-day follow-up but doesn't

Major problems (+5 points per problem)
High levels of missingness
High levels of unknowns

medRxiv preprint doi: https://doi.org/10.1101/2023.03.09.23287038; this version posted March 10, 2023. The copyright holder for this preprint (which was not certified by peer review) is the author/funder, who has granted medRxiv a license to display the preprint in perpetuity. It is made available under a CC-BY 4.0 International license .

APPENDIX B: Breast Cancer Disparities Statistical Analysis Plan:

Approved Project Title: **Racial and Ethnic Disparities among Patients with Breast Cancer and COVID-19 in CCC19 Cohort**

Project Team Leads: Gayathri Nagaraj, Melissa Accordino, Maryam Lustberg, Dimpy Shah

Name of the investigator completing this survey: Gayathri Nagaraj and Melissa Accordino

Proposed milestone deadline for this manuscript:

- Abstract submission for ASCO 2021, deadline February 17 completed.
- ASCO abstract accepted for oral presentation. Deadlines for prelim slide upload May 7, and final deadline for uploading slides May 14.
- Manuscript preparation simultaneously, deadline and journal TBD

Do you have local statistical support: No

Name and emails of (at most) 2 additional project team members who would like to be part of the analysis team for the project:

Melissa Accordino, Email: mkg2134@cumc.columbia.edu

Maryam Lustberg, Email: Maryam.Lustberg@osumc.edu

Dimpy Shah, Email: shahdp@uthscsa.edu

Initial draft of the Statistical Analysis Plan (SAP), following STROBE guidelines, for our review and input. Please complete sections 1 and 3-11 (and 12 if you have local statistical support)

1 (a) Manuscript Title: **Racial and Ethnic Disparities among Patients with Breast Cancer and COVID-19 in CCC19 Cohort**

1 (b) Provide in the abstract an informative and balanced summary of what was done and what will be found.

Racial and Ethnic minority subgroups are at a disproportionately increased risk of contracting COVID-19 or experiencing severe illness regardless of age. Racial and Ethnic disparities also affect breast cancer incidence and mortality. The impact of COVID-19 on patients with breast cancer is largely unknown but is currently under investigation. Outcomes of COVID-19 specifically in racial and ethnic minority patients with active or prior history of breast cancer is currently unknown.

medRxiv preprint doi: https://doi.org/10.1101/2023.03.09.23287038; this version posted March 10, 2023. The copyright holder for this preprint (which was not certified by peer review) is the author/funder, who has granted medRxiv a license to display the preprint in perpetuity.
It is made available under a CC-BY 4.0 International license .

3. Objectives

State specific objectives, including any prespecified hypotheses:

<u>The overarching goal</u> of this study is to evaluate the racial and ethnic disparities related to COVID-19 outcomes, in patients with active or previous history of breast cancer. To evaluate this, the following specific aims are proposed:

- **<u>Specific Aim 1:</u> To compare the distribution of major clinical, sociodemographic, and breast cancer risk factors among racial and ethnic subgroups of women with active or previous history of single primary invasive breast cancer diagnosed with COVID-19.**
  We *<u>hypothesize</u>* that racial and ethnic minority women with breast cancer are more likely to have active comorbid conditions, such as diabetes mellitus, obesity, smoking history, and a baseline lower performance status compared to non-Hispanic white (NHW) women with active or previous history of breast cancer diagnosed with COVID-19. Other variables of interest are age, month/year of COVID-19 diagnosis, area of patient residence, geographic region, insurance type, treatment center characteristics, receipt of anti-COVID-19 treatment along with tumor characteristics including breast cancer biologic subtype, cancer status, treatment intent, timing of anti-cancer treatment and modality of anti-cancer treatment.

- **<u>Specific Aim 2:</u> To compare COVID-19 clinical outcomes on a five-level ordinal scale based on patient's most severe reported outcomes: no complications (uncomplicated); hospital admission, intensive care unit (ICU) admission, mechanical ventilation; or death from any cause in racial and ethnic minority subgroups of women with previous or active history of breast cancer compared to NHW adjusted for baseline characteristics. We also plan to evaluate the death within 30 days of COVID-19 diagnosis among racial and ethnic subgroups of women with previous or active history of breast cancer compared to NHW adjusted for baseline characteristics.**
  We *<u>hypothesize</u>* that there will be higher rates of severe COVID-19 related outcomes in the racial and ethnic minority subgroups compared to non-Hispanic white (NHW) patients with active or previous history of breast cancer.

- **<u>Exploratory aims:</u>**

  1. To evaluate the frequency of hospitalization, supplemental oxygen use, ICU admission and use of mechanical ventilation in the various racial ethnic groups**.**
  2. To describe the distribution of major clinical, sociodemographic, breast cancer risk factors and outcomes in men with active or previous history of breast cancer diagnosed with COVID-19.
  3. Assess the rate of major clinical complications such as cardiovascular, pulmonary, gastrointestinal, superimposed infection, vascular thrombosis and others among various racial and ethnic groups of women with active or previous history of breast cancer.

4. Study Design

Study Design:

Present key elements of study design early in the paper

This is a retrospective cohort study using de-identified data from the COVID-19 and Cancer Consortium (CCC19) database which is a centralized multi-institution registry of patients with current or past history of cancer diagnosed with COVID-19. Study data are collected and managed using REDCap software hosted at Vanderbilt University Medical Center.

medRxiv preprint doi: https://doi.org/10.1101/2023.03.09.23287038; this version posted March 10, 2023. The copyright holder for this preprint (which was not certified by peer review) is the author/funder, who has granted medRxiv a license to display the preprint in perpetuity.
It is made available under a CC-BY 4.0 International license .

5. Setting

Setting

Describe the setting, locations, and relevant dates, including periods of recruitment, exposure, follow-up, and data collection.

The CCC19 international registry consists of de-identified data on adult patients (18 years and older) with a current or past history of hematologic malignancy or invasive solid tumor who either have laboratory-confirmed SARS-CoV-2 infection or presumptive diagnosis of COVID-19. The CCC19 registry includes patients with either active cancer or a history of cancer and contains variables related to patient demographics, cancer history, and COVID-19 clinical course including receipt of COVID-19 related therapeutics along with follow-up data. The member institutions of the consortium report data through the online REDCap data collection survey developed by CCC19. Data collection period is ongoing, for the purpose of this analysis, the data collected from March 17, 2020 to February 9’ 2021 will be used.

6. Participants

(a) Give the eligibility criteria, and the sources and methods of selection of participants. Describe methods of follow-up:

Patients with active or previous history of invasive breast cancer with evaluable self-reported race/ethnicity data, and with laboratory confirmed COVID-19 will be our study population. While the primary analysis will be restricted to women with active or previous history of breast cancer and descriptive data on men with active or previous history of breast cancer will be provided separately as part of the exploratory analysis given the small numbers. We will restrict our analysis to patients diagnosed in the United States of America since the racial and ethnic disparities of interest have been previously described in United States. We will also exclude patients who have multiple malignancies including a history of bilateral breast cancer with the exception of contralateral DCIS only. Further, patients who are not evaluable for the primary ordinal outcome or with a data quality score >4 will be excluded. For this analysis, the unknown/Not reported category of race and ethnicity will be excluded.

medRxiv preprint doi: https://doi.org/10.1101/2023.03.09.23287038; this version posted March 10, 2023. The copyright holder for this preprint (which was not certified by peer review) is the author/funder, who has granted medRxiv a license to display the preprint in perpetuity.
It is made available under a CC-BY 4.0 International license .

CONSORT flow diagram

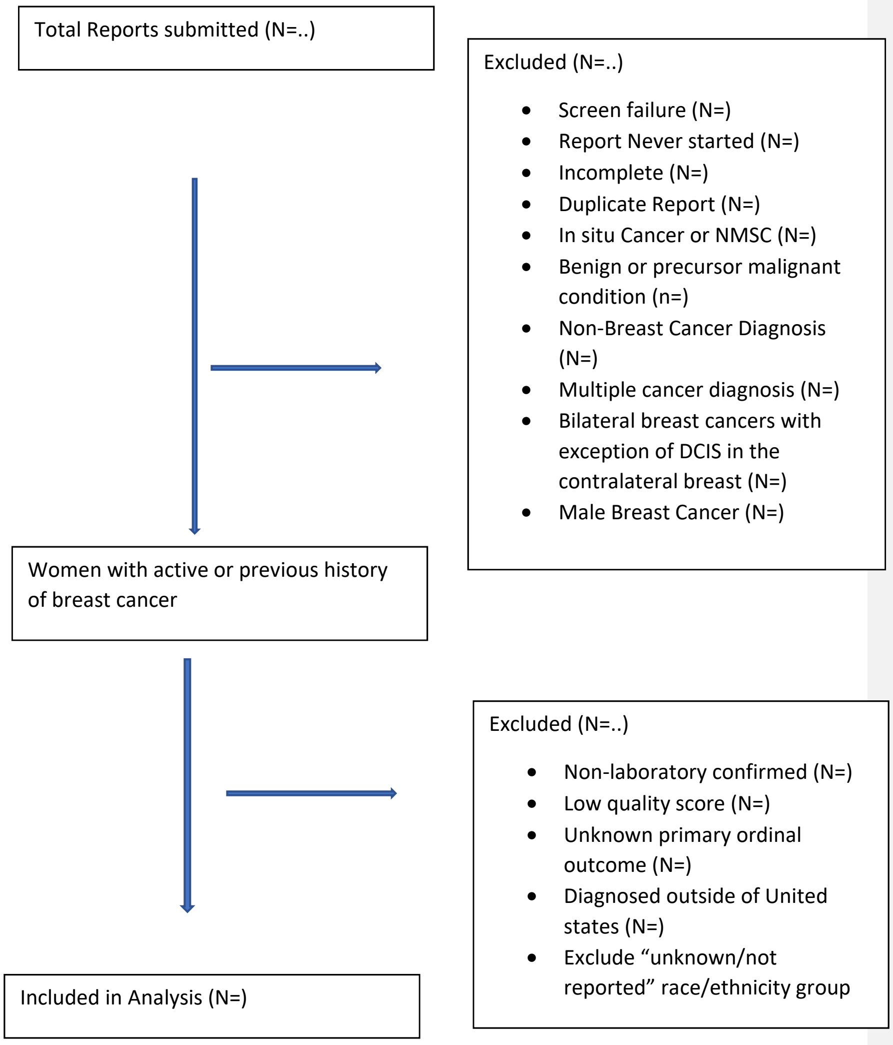

medRxiv preprint doi: https://doi.org/10.1101/2023.03.09.23287038; this version posted March 10, 2023. The copyright holder for this preprint (which was not certified by peer review) is the author/funder, who has granted medRxiv a license to display the preprint in perpetuity.
It is made available under a CC-BY 4.0 International license .

(b) For matched studies, give matching criteria and number of exposed and unexposed:

Not applicable as the CCC19 registry does not carry data for cancer patients who are not exposed to Covid-19.

7. Variables (Clearly define all variables)

Outcomes:

- Primary: COVID-19 severity outcome defined on a five-level ordinal scale based on patient's most severe reported outcomes: No complications (uncomplicated); hospital admission; intensive care unit admission, mechanical ventilation; or death from any cause.
- Secondary: 30-day all-cause mortality
- Exploratory/Descriptive:
    - Rates of hospitalization; oxygen requirements; ICU admission; mechanical ventilation.
    - Major clinical complications (cardiovascular, pulmonary, gastrointestinal, AKI, MOF, superimposed infection, sepsis, any bleeding, DIC, Thrombosis).
    - Descriptive statistics for men with breast cancer diagnosed with COVID-19.

Exposures

Predictors

1) Self-reported race
2) Self-reported ethnicity

Potential confounders

Higher priority

1) Age in years
2) Obesity (obese, not obese)
3) Co-morbidities (pulmonary, cardiovascular, renal, diabetes mellitus)
4) ECOG PS (0, 1, ≥2, unknown)
5) Receptor status (Hormone receptor positive, HER2 positive, dual positive, Triple negative)
6) Cancer status (remission < 5 years, remission > 5 years, active stable, active responding, active progressing, unknown)
7) Timing of anti-cancer treatment (never treated, 0-4 weeks, 1-3 months, >3 months)
8) Modality of recent anti-cancer treatment (none, cytotoxic chemotherapy, targeted therapy, endocrine therapy, immunotherapy, locoregional therapy, other)
9) Period of COVID-19 diagnosis (Jan-April 2020, May-August 2020, Sep-Nov 2020, Dec 2020-Feb 2021)

<u>Lower priority</u>

10) Smoking (ever, never)
11) US region of patient residence (NE, MW, South, West)
12) Area of patient residence (urban, suburban, rural)
13) Insurance status (Not insured, private insurance, Medicaid/Medicare, other government, missing/unknown)
14) Treatment center characteristics academic (university, tertiary and NCI designated comprehensive cancer centers), community (practice and hospital), other.

Effect modifiers
None

medRxiv preprint doi: https://doi.org/10.1101/2023.03.09.23287038; this version posted March 10, 2023. The copyright holder for this preprint (which was not certified by peer review) is the author/funder, who has granted medRxiv a license to display the preprint in perpetuity.
It is made available under a CC-BY 4.0 International license .

Diagnostic criteria (if applicable)

8. Data Sources / Measurement

For each variable of interest, give sources of data and details of methods of assessment (measurement). Describe comparability of assessment methods if there is more than one group.

9. Bias

Describe any efforts to address potential sources of bias

Multivariable regression models will be used to adjust for known confounding variables.

10. Study size

Explain how the study size was arrived at

Study size is based on the number of breast cancer cases reported in the registry at the time of Analysis. Breast cancer is the single largest solid tumor cohort within the CCC19 registry accounting for roughly 21% of cases. The numbers are expected to rise given the steep accrual rate.

11. Quantitative variables

Explain how quantitative variables will be handled in the analyses. If applicable, describe which groupings will be chosen and why

12. Statistical methods

(a) Describe all statistical methods, including those to be used to control for confounding

Primary analysis among women:

Standard descriptive statistics will summarize major clinical, demographic, and breast cancer prognostic factors; clinical complications during hospitalization; and rates of 30-day mortality, hospitalization, oxygen requirement, ICU admission, and mechanical ventilation among racial and ethnic subgroups. Multivariable ordinal and binary logistic regression models will estimate differences in adjusted odds of COVID-19 severity and 30-day mortality, respectively, between racial and ethnic subgroups. Because the ordinal outcome is assessed over patient's total follow-up period, the model will include an offset for (log) follow-up time. Adjustment covariates will be selected first from the "higher priority" confounders listed above, followed by those listed as "lower priority." Coefficients and standard errors from models with different levels of adjustment, variance inflation factors, and clinical judgement will be used to assess model stability.

Descriptive analysis among men:

We will calculate standard descriptive statistics for major clinical, demographic, and breast cancer prognostic factors and clinical complications during hospitalization; rates of 30-day mortality, hospitalization, oxygen requirement, ICU admission, and mechanical ventilation among men with active or previous history of breast cancer.

medRxiv preprint doi: https://doi.org/10.1101/2023.03.09.23287038; this version posted March 10, 2023. The copyright holder for this preprint (which was not certified by peer review) is the author/funder, who has granted medRxiv a license to display the preprint in perpetuity.
It is made available under a CC-BY 4.0 International license .

(b) Describe any methods that will be used to examine subgroups and interactions

None included.

(c) Explain how missing data will be addressed

Multiple imputation will be used to impute missing and unknown data for all variables included in the analysis, with some exceptions: unknown ECOG performance score and unknown cancer status will not be imputed and treated as a separate category in analyses. Imputation will be performed on the largest dataset possible (that is, after removing test cases and other manual exclusions, but before applying specific exclusion criteria). At least 10 imputed datasets will be used.

(d) If applicable, explain how loss to follow-up will be addressed

All observed outcomes will be used with models adjusted for duration of follow-up.

(e) Describe any sensitivity analyses

None.

medRxiv preprint doi: https://doi.org/10.1101/2023.03.09.23287038; this version posted March 10, 2023. The copyright holder for this preprint (which was not certified by peer review) is the author/funder, who has granted medRxiv a license to display the preprint in perpetuity.
It is made available under a CC-BY 4.0 International license .

APPENDIX C: CCC19 Approved Project Variables

Primary Outcome (Table III)

Outcome description	Outcome variable name	Outcome values
**Custom ordinal outcome with death at any time**	*der_ordinal_v1a*	0 = not hospitalized; 1 = hospitalized; 2 = ICU; 3 = mechanical ventilation; 4 = death at any time
**Follow-up in days, with some estimation for intervals**	*der_days_fu*	Integer (days)

Secondary Outcome (Table II)

Outcome description	Outcome variable name	Outcome values	Additional Details
**Derived dead/alive variable**	*der_deadbinary*	0 = No; 1 = Yes; 99 = Unknown	
**Derived variable indicating whether patient has died within 30 days of COVID-19 diagnosis (default = No)**	der_dead30	0 = No; 1 = Yes; 99 = Unknown	
**Derived variable indicating whether patients required mechanical ventilation**	der_mv	0 = No; 1 = Yes; 99 = Unknown	
**Derived variable indicating time in ICU**	der_ICU	0 = No; 1 = Yes; 99 = Unknown	
**Derived hospitalized/not hospitalized variable**	der_hosp	0 = No; 1 = Yes; 99 = Unknown	
**Derived cardiovascular complication variable (see additional details)**	der_CV_event_v2 (der_any_CV is the variable name in R script)	0 = No; 1 = Yes; 99=Unknown.	Derived with the following derived variables:<br><br>der_hotn_comp, der_MI_comp, der_card_isch_comp, der_AFib_comp, der_VF_comp, der_arry_oth_comp, der_CMY_comp, der_CHF_comp, der_PE_comp, der_DVT_comp, der_stroke_comp, der_thrombosis_NOS_comp<br><br>Coded as 1 if any of these variables is 1; coded as 0 if all these variables are 0; coded as 99 if any of variables is 99 and der_CV_event_v2 is missing;

medRxiv preprint doi: https://doi.org/10.1101/2023.03.09.23287038; this version posted March 10, 2023. The copyright holder for this preprint (which was not certified by peer review) is the author/funder, who has granted medRxiv a license to display the preprint in perpetuity.
It is made available under a CC-BY 4.0 International license .

			otherwise, NA<br><br>For all listed variable here:<br>0=No, 1=Yes, 99=Unknown
**Derived pulmonary complication variable (see additional details)**	der_pulm_event (der_any_Pulm is the variable name in R script)	0 = No; 1 = Yes; 99=Unknown.	Derived with the following derived variables:<br><br>der_resp_failure_comp, der_pneumonitis_comp, der_pneumonia_comp, der_ARDS_comp, der_PE_comp, der_pleural_eff_comp, der_empyema_comp<br><br>Coded as 1 if any of these variables is 1; coded as 0 if all these variables are 0; coded as 99 if any of variables is 99 and der_pulm_event is missing; otherwise, NA<br><br>For all listed variable here:<br>0=No, 1=Yes, 99=Unknown
**Derived gastrointestinal complication variable (see additional details)**	der_GI_event (der_any_Gast is the variable name in R script)	0 = No; 1 = Yes; 99=Unknown.	Derived with the following derived variables:<br><br>der_AHI_comp, der_ascites_comp, der_BO_comp, der_bowelPerf_comp, der_ileus_comp, der_peritonitis_comp<br><br>Coded as 1 if any of these variables is 1; coded as 0 if all these variables are 0; coded as 99 if any of variables is 99 and der_GI_event is missing; otherwise, NA<br><br>For all listed variable here:<br>0=No, 1=Yes, 99=Unknown
**Acute kidney injury (checkbox only)**	der_AKI_comp	0 = No; 1 = Yes; 99 = Unknown	
**Multisystem organ failure**	der_MOF_comp	0 = No; 1 = Yes; 99 = Unknown	
**Any co-infection within +/- 2 weeks of COVID-19 dx**	der_coinfection_any	0 = No; 1 = Yes; 99 = Unknown	
**Sepsis**	der_sepsis_comp	0 = No; 1 = Yes; 99 = Unknown	
**Bleeding**	der_bleeding_comp	0 = No; 1 = Yes; 99 = Unknown	
**DIC (without modifier of**	der_DIC_comp	0 = No; 1 = Yes; 99 =	

medRxiv preprint doi: https://doi.org/10.1101/2023.03.09.23287038; this version posted March 10, 2023. The copyright holder for this preprint (which was not certified by peer review) is the author/funder, who has granted medRxiv a license to display the preprint in perpetuity.
It is made available under a CC-BY 4.0 International license .

**definite/probable/possible)**		Unknown	
**Remdesivir as treatment for COVID-19 ever**	der_rem	0 = No; 1 = Yes; 99 = Unknown	
**Hydroxychloroquine as COVID-19 treatment ever**	der_hcq	0 = No; 1 = Yes; 99 = Unknown	
**Steroids as COVID-19 treatment ever**	der_steroids_c19	0 = No; 1 = Yes; 99 = Unknown	
**COVID-19 treatments other than HCQ, steroids, remdesivir**	der_other_tx_c19_v2	0 = No; 1 = Yes; 99 = Unknown	
**Indicates whether patient has ever had supplemental o2**	der_o2_ever	0 = No; 1 = Yes; 99 = Unknown	

Covariate description	Variable name	Covariate values	Additional Details
**Race/ethnicity including Asian**	*der_race_v2*	Hispanic; Non-Hispanic AAPI; Non-Hispanic Black; Non-Hispanic White; Other	
**Age with imputation for categoricals**	*der_age_trunc*	Years (continuous 18-89; patients noted to be greater than 89 are set to be age = 90)	
**Insurance type**	der_insurance	Medicaid alone; Medicare alone; Medicare/Medicaid +/- other; Other government +/- other; Private +/- other; Uninsured; Unknown	
**Derived variable for smoking status collapsing the current/former smoker variables**	der_smoking2	Never; Current or Former; Unknown	
**Binary obesity (BMI >= 30 or checkbox checked) indicator**	der_obesity	0 = No; 1 = Yes; 99 = Unknown	
**Cardiovascular comorbidity (CAD, CHF, Afib, arrhythmia NOS, PVD, CVA, cardiac disease NOS)**	der_card	0 = No; 1 = Yes; 99 = Unknown	
**Derived variable indicating whether patient has pulmonary comorbidities**	der_pulm	0 = No; 1 = Yes; 99 = Unknown	
**Renal comorbidities**	der_renal	0 = No; 1 = Yes; 99 =	

medRxiv preprint doi: https://doi.org/10.1101/2023.03.09.23287038; this version posted March 10, 2023. The copyright holder for this preprint (which was not certified by peer review) is the author/funder, who has granted medRxiv a license to display the preprint in perpetuity.
It is made available under a CC-BY 4.0 International license .

		Unknown	
**Derived variable indicating whether patient has diabetes mellitus**	der_dm2	0 = No; 1 = Yes; 99 = Unknown	
**Performance Status**	der_ecogcat2	ECOG 0, 1, or 2+	
**Breast biomarkers combined variable**	der_breast_biomarkers	1 = ER+; 2 = ER+/HER2+; 3 = HER2+; 4 = triple negative; 99 = Unknown	
**Derived variable indicating cancer status (Splits remission/NED by cancer timing)**	der_cancer_status_v4	0 - Remission/NED, remote; 1 - Remission/NED, recent; 2 - Active, responding; 3 - Active, stable; 4 - Active, progressing; 99 - Unknown	
**Timing of cancer treatment relative to COVID-19, collapsed**	der_cancer_tx_timing_v2	0 = more than 3 months; 1 = 0-4 weeks; 2 = 1-3 months (*); 88 = never or after COVID-19 diagnosis; 99 = unknown	
**No cancer treatment in the 3 months prior to COVID-19**	der_cancertr_none	0=No; 1=Yes; 99=Unknown	Derived with the following covariates:<br><br>der_any_cyto, der_any_targeted, der_any_endo, der_any_immuno, der_any_local, der_any_other<br><br>Coded as 1 if all these variables are 0; coded as 0 if any of these variables is 1; coded as 99 if any of these variables is 99; otherwise, NA
**Any cytotoxic cancer treatment in the 3 months prior to COVID-19**	der_any_cyto	0 = No; 1 = Yes; 99 = Unknown	
**Any targeted therapy in the 3 months prior to COVID-19**	der_any_targeted	0 = No; 1 = Yes; 99 = Unknown	
**Any targeted therapy includes an anti-HER2 therapy in the 3 months prior to COVID-19**	der_her2_3m	0 = No; 1 = Yes	Derived with der_her2, der_any_targeted.<br><br>Coded as 1 if der_any_targeted is 1 and der_her2 is 1<br><br>Coded as 0 if:<br>a. der_any_targeted is 1 and der_her2 is 0<br>b. der_any_targeted is 1<br><br>Otherwise, NA

medRxiv preprint doi: https://doi.org/10.1101/2023.03.09.23287038; this version posted March 10, 2023. The copyright holder for this preprint (which was not certified by peer review) is the author/funder, who has granted medRxiv a license to display the preprint in perpetuity.
It is made available under a CC-BY 4.0 International license .

			der_her2:<br>0 = No; 1 = Yes
**Any targeted therapy includes a CDK4/6 inhibitor therapy in the 3 months prior to COVID-19**	der_cdk46i_3m	0 = No; 1 = Yes	Derived with der_cdk46i, der_any_targeted.<br><br>Coded as 1 if der_any_targeted is 1 and der_cdk46i is 1<br><br>Coded as 0 if:<br>a. der_any_targeted is 1 and der_cdk46i is 0<br>b. der_any_targeted is 1<br><br>Otherwise, NA<br><br>der_cdk46i:<br>0 = No; 1 = Yes
**Any other targeted therapy (Not anti-HER2 / CDK4/6 inhibitor) in the 3 months prior to COVID-19**	der_other_3m	0 = No; 1 = Yes	Derived with der_targeted_not_her2_cdk46i, der_any_targeted.<br><br>Coded as 1 if der_any_targeted is 1 and der_targeted_not_her2_cdk46i is 1<br><br>Coded as 0 if:<br>a. der_any_targeted is 1 and der_targeted_not_her2_cdk46i is 0<br>b. der_any_targeted is 1<br><br>Otherwise, NA<br><br>der_targeted_not_her2_cdk46i:<br>0 = No; 1 = Yes
**Any endocrine therapy in the 3 months prior to COVID-19**	der_any_endo	0 = No; 1 = Yes; 99 = Unknown	
**Any immunotherapy in the 3 months prior to COVID-19**	der_any_immuno	0 = No; 1 = Yes; 99 = Unknown	
**Any local therapy (surgery or RT) within 3 months**	der_any_local	0 = No; 1 = Yes; 99 = Unknown	
**Any other cancer therapy in the 3 months prior to COVID-19**	der_any_other	0 = No; 1 = Yes; 99 = Unknown	
**Region of patient residence with ex-US collapsed**	der_region_v2	Non-US; Other; Undesignated US; US Midwest; US Northeast; US South; US West	
**Trimester and year of**	der_tri_rt_dx	T1 2020; T2 2020; T3	

medRxiv preprint doi: https://doi.org/10.1101/2023.03.09.23287038; this version posted March 10, 2023. The copyright holder for this preprint (which was not certified by peer review) is the author/funder, who has granted medRxiv a license to display the preprint in perpetuity.
It is made available under a CC-BY 4.0 International license .

**diagnosis, using the most recent side of the interval as anchor**		2020; T1 2021	
**What type of area does the patient primarily reside in?**	urban_rural[1]	1, Urban (city) | 2, Suburban (town, suburbs) | 3, Rural (country) | 88, Other | 99, Unknown	
**The type of health care center providing the patient's data**	der_site_type	AMC = academic medical center; CP = community practice; TCC = tertiary care center	
**Initial Severity and Course of Illness**	severity_of_covid_19_v2[1]	1, Mild (no hospitalization required) | 2, Moderate (hospitalization indicated) | 3, Severe (ICU admission indicated) | 99, Unknown	
**Derived treatment intent**	der_tr_intent	Unknown Treatment; Not on Treatment; Palliative; Curative; Missing<br><br>Unknown Treatment and Missing were collapsed for analysis	Derived with der_anytx and treatment_intent:<br><br>Coded as "Unknown Treatment" if der_anytx is NA or 99;<br><br>Coded as "Not on Treatment" if der_anytx is 0<br><br>Coded as "Palliative" if der_anytx is 1 and treatment_intent is 2<br><br>Coded as "Curative" if der_anytx is 1 and treatment_intent is 1<br><br>Otherwise, Missing<br><br>der_anytx:<br>0 = No; 1 = Yes; 99 = Unknown<br><br>Treatment_intent: 1, Curative | 2, Palliative | 99, Unclear or unknown
**Most recent line of cancer treatment, including systemic and non-systemic therapies**	der_txline	Untreated in last 12 months; Curative NOS; First line; Non-curative NOS; Other; Second line or greater; Unknown	
**Hematologic malignancy indicator**	der_heme	0 = No; 1 = Yes	

901

Other covariate related to cohort selection for analysis	Variable name	Covariate values	Covariate description
***Sex* (Recode other/prefer not to**	*der_sex*	*Male, Female*	

medRxiv preprint doi: https://doi.org/10.1101/2023.03.09.23287038; this version posted March 10, 2023. The copyright holder for this preprint (which was not certified by peer review) is the author/funder, who has granted medRxiv a license to display the preprint in perpetuity.
It is made available under a CC-BY 4.0 International license .

**say gender --> missing*)***			
**Breast cancer**	der_Breast	0 = No; 1 = Yes	
**Cancer type of second malignancy.**<br><br>**If the patient has more than two malignancies, please select the second-most recently diagnosed cancer type. If unknown or unclear, please specify in the free text box below**	cancer_type_2[1]	‘’ indicates no second malignancy.	
**Region of patient residence with US and ex-US collapsed**	der_region_v3	Non-US; Other; US	

New covariate request – 2-5-22

New covariate	Variable name	Covariate values	Covariate description
**MBC vs non-MBC**	der_metastatic	*0 = No; 1 = Yes; 99 = Unknown*	*Metastatic cancer status (only applicable to solid tumors/lymphoma)*
**MBC site of metastasis**	der_met_bone	0 = No; 1 = Yes; 99 = Unknown	Metastatic to bone
**MBC site of metastasis**	der_met_liver	0 = No; 1 = Yes; 99 = Unknown	Metastatic to liver
**MBC site of metastasis**	der_met_lung_v2	0 = No; 1 = Yes; 99 = Unknown	Metastatic to lung

medRxiv preprint doi: https://doi.org/10.1101/2023.03.09.23287038; this version posted March 10, 2023. The copyright holder for this preprint (which was not certified by peer review) is the author/funder, who has granted medRxiv a license to display the preprint in perpetuity.
It is made available under a CC-BY 4.0 International license .

APPENDIX D: Supplementary Figure I - Proportional Odds Assumption

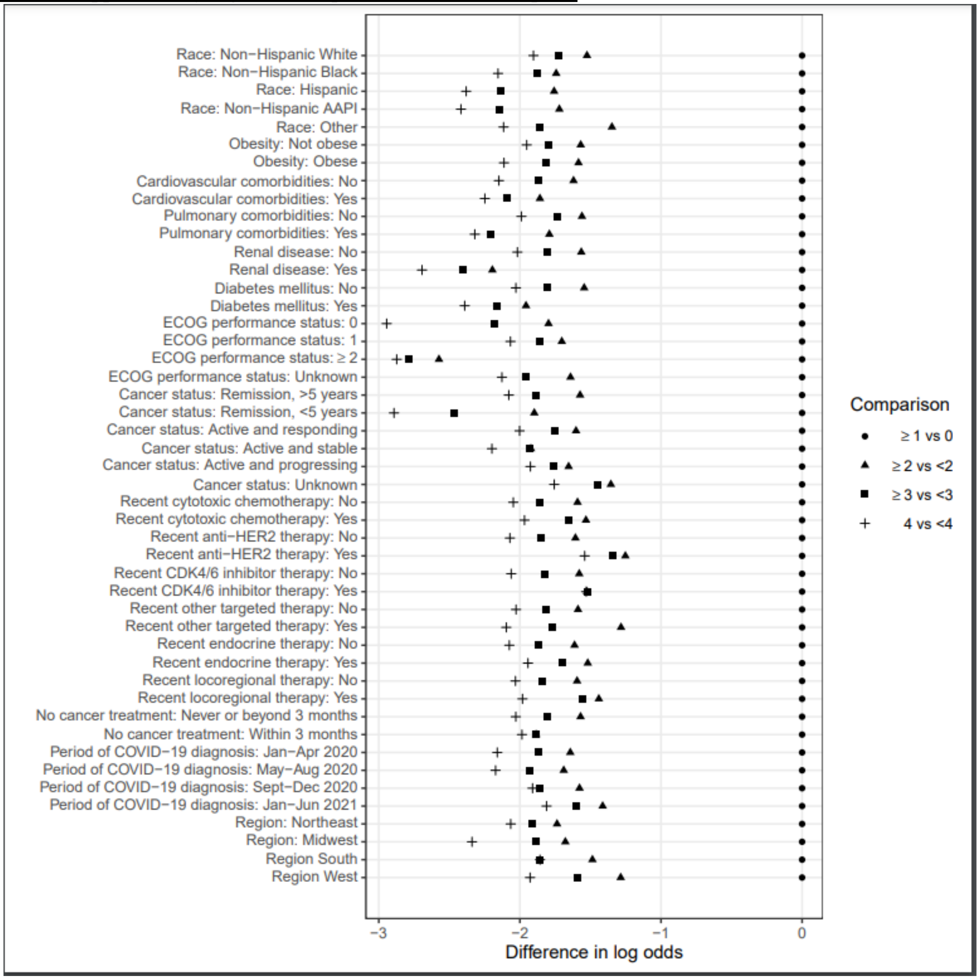

medRxiv preprint doi: https://doi.org/10.1101/2023.03.09.23287038; this version posted March 10, 2023. The copyright holder for this preprint (which was not certified by peer review) is the author/funder, who has granted medRxiv a license to display the preprint in perpetuity.
It is made available under a CC-BY 4.0 International license .

APPENDIX E

Supplement Table 1. Unadjusted rates of outcomes after COVID-19 diagnosis by cancer status

	NED>5 Years	NED<5 Years	Active and Responding	Active and Stable	Active and Progressing	Missing/ Unknown	Total
	n[a] (%)	n[a] (%)	n[a] (%)	n[a] (%)	n[a] (%)	n[a] (%)	n[a] (%)
Outcomes							
Total all-cause mortality [b]	40 (11)	12 (3)	12 (7)	11 (7)	37 (38)	11 (9)	123 (9)
30-day all-cause mortality [c]	29 (8)	10 (2)	10 (6)	4 (2)	27 (28)	9 (7)	89 (6)
Received mechanical ventilation [b]	20 (5)	13 (3)	9 (5)	7 (4)	12 (12)	8 (7)	69 (5)
Admitted to an intensive care unit [b]	35 (10)	25 (6)	13 (8)	8 (5)	18 (19)	12 (10)	111 (8)
Admitted to the hospital [b]	163 (43)	129 (29)	54 (31)	57 (34)	70 (72)	39 (32)	512 (37)

[a]N is based on non-missing data

[b]Included in primary ordinal COVID-19 severity outcome.

[c]Secondary outcome.

medRxiv preprint doi: https://doi.org/10.1101/2023.03.09.23287038; this version posted March 10, 2023. The copyright holder for this preprint (which was not certified by peer review) is the author/funder, who has granted medRxiv a license to display the preprint in perpetuity.
It is made available under a CC-BY 4.0 International license .

APPENDIX F

Supplementary Table IIA: Baseline characteristics of female patients with MBC

	**MBC (N=233)**
**Age, years[a]**	
Median [IQR]	58.0 [49.8, 68.3]
**Race/Ethnicity**	
Non-Hispanic White	107 (46%)
Non-Hispanic Black	56 (24%)
Hispanic	50 (21%)
Non-Hispanic AAPI	10 (4%)
Other	10 (4%)
**Smoking Status**	
Never	162 (70%)
Current or Former	66 (28%)
Missing/unknown	5 (2%)
**Obesity**	
No	139 (60%)
Yes	93 (40%)
**Comorbidities[b]**	
Cardiovascular	42 (18%)
Pulmonary	37 (16%)
Renal Disease	16 (7%)
Diabetes mellitus	52 (22%)
Missing/unknown	3 (1%)
**ECOG Performance Status**	
0	63 (27%)
1	84 (36%)
2+	42 (18%)
Unknown	44 (19%)
Missing	0 (0%)
**Receptor status**	
HR+/HER2-	98 (42%)
HR+/HER2+	53 (23%)
HR-/HER2+	26 (11%)
Triple Negative	33 (14%)
Missing/unknown	23 (10%)
**Cancer Status**	

medRxiv preprint doi: https://doi.org/10.1101/2023.03.09.23287038; this version posted March 10, 2023. The copyright holder for this preprint (which was not certified by peer review) is the author/funder, who has granted medRxiv a license to display the preprint in perpetuity.
It is made available under a CC-BY 4.0 International license .

Active and responding	55 (24%)
Active and stable	78 (33%)
Active and progressing	74 (32%)
Unknown	25 (11%)
Missing	0 (0%)
**Metastatic sites (MBC)**	
Lung	65 (28%)
Bone	135 (58%)
Liver	61 (26%)
Missing/unknown	19 (8%)
**Timing of anti-cancer therapy**	
Never/After COVID-19	X*
0-4 weeks	189 (81%)
1-3 months	14 (6%)
>3 months	19 (8%)
Missing/unknown	11 (5%)*
**Modality of active anti-cancer therapy**[b,c]	
None	24 (10%)
Cytotoxic Chemotherapy	114 (49%)
Targeted Therapy	115 (49%)
Endocrine Therapy	98 (42%)
Immunotherapy	17 (7%)
Local (Surgery/Radiation)	27 (12%)
Other	6 (3%)
Missing/unknown	6 (3%)
**Region**	
Northeast	97 (42%)
Midwest	44 (19%)
South	34 (15%)
West	56 (24%)
Undesignated	2 (1%)
**Period of COVID-19 diagnosis**	
Jan-Apr 2020	33 (14%)
May-Aug 2020	101 (43%)
Sept-Dec 2020	52 (22%)
Jan-Aug 2021	45 (19%)
Missing/unknown	2 (1%)
**Area of patient residence**	
Urban	103 (44%)
Suburban	80 (34%)
Rural	12 (5%)
Missing/unknown	38 (16%)
**Treatment center characteristics**	

medRxiv preprint doi: https://doi.org/10.1101/2023.03.09.23287038; this version posted March 10, 2023. The copyright holder for this preprint (which was not certified by peer review) is the author/funder, who has granted medRxiv a license to display the preprint in perpetuity.
It is made available under a CC-BY 4.0 International license .

Academic Medical Center	43 (18%)
Community Practice	63 (27%)
Tertiary Care Center	127 (55%)
Missing/unknown	0 (0%)
**Severity of COVID19**	
Mild	126 (54%)
Moderate	93 (40%)
Severe	13 (6%)
Missing/Unknown	1 (<1%)
***Cells combined to mask N<5 according to CCC19 low count policy**	

[a]Age was truncated at 90 years.

[b]Percentages could sum to >100% because categories are not mutually exclusive.

[c]Within 3 months of COVID-19 diagnosis.

938

Supplement Table IIB: Unadjusted rates of outcomes after COVID-19 diagnosis in females with MBC

medRxiv preprint doi: https://doi.org/10.1101/2023.03.09.23287038; this version posted March 10, 2023. The copyright holder for this preprint (which was not certified by peer review) is the author/funder, who has granted medRxiv a license to display the preprint in perpetuity.
It is made available under a CC-BY 4.0 International license .

	n[f] (%)
Outcomes	
Total all-cause mortality [a]	45 (19)
30-day all-cause mortality [b]	28 (12)
Received mechanical ventilation [a]	20 (9)
Admitted to an intensive care unit [a]	29 (12)
Admitted to the hospital [a]	124 (53)
Clinical Complications	
Any cardiovascular complication [c]	48 (21)
Any pulmonary complication [d]	86 (37)
Any gastrointestinal complication [e]	13 (6)
Acute kidney injury	32 (14)
Multisystem organ failure	12 (5)
Superimposed infection	32 (14)
Sepsis	28 (12)
Any bleeding	8 (3)
Interventions	
Remdesivir	35 (15)
Hydroxychloroquine	25 (11)
Corticosteroids	65 (29)
Covid Other	45 (20)
Supplemental oxygen	84 (37)

[a]Included in primary ordinal COVID-19 severity outcome

[b]Secondary outcome

[c]Cardiovascular complication includes hypotension, myocardial infarction, other cardiac ischemia, atrial fibrillation, ventricular fibrillation, other cardiac arrhythmia, cardiomyopathy, congestive heart failure, pulmonary embolism (PE), deep vein thrombosis (DVT), stroke, thrombosis NOS complication.

[d]Pulmonary complication includes respiratory failure, pneumonitis, pneumonia, acute respiratory distress syndrome (ARDS), PE, pleural effusion, empyema.

[e]Gastrointestinal complication includes acute hepatic injury, ascites, bowel obstruction, bowel perforation, ileus, peritonitis

[f]N is based on non-missing data

APPENDIX G

medRxiv preprint doi: https://doi.org/10.1101/2023.03.09.23287038; this version posted March 10, 2023. The copyright holder for this preprint (which was not certified by peer review) is the author/funder, who has granted medRxiv a license to display the preprint in perpetuity.
It is made available under a CC-BY 4.0 International license .

Supplement Table III: Adjusted Associations of Race Factors with COVID-19 Severity Outcome.

	COVID-19 severity		
	OR (95% CI)	Point Value E estimates[a]	Lower bound E values[a]
**Race (Ref: NHW)**			
Black	1.74 (1.24- 2.45)	1.97	1.47
Hispanic	1.38 (0.93- 2.05)	1.63	1.00
AAPI	3.40 (1.70- 6.79)	3.09	1.93
Other	2.97 (1.71- 5.17)	2.84	1.94

[a] These values were calculated based on the formula for logistic regression

APPENDIX H:

medRxiv preprint doi: https://doi.org/10.1101/2023.03.09.23287038; this version posted March 10, 2023. The copyright holder for this preprint
(which was not certified by peer review) is the author/funder, who has granted medRxiv a license to display the preprint in perpetuity.
It is made available under a CC-BY 4.0 International license .

**Supplement Table IVA: Male patients with breast cancer and COVID-19: Baseline Characteristics**	
**Total**	**25 (100%)**
**Age, years**[a]	
Median [IQR]	67.0 [60 - 75]
**Race/Ethnicity**	
NHW	13 (52%)
Black	8 (32%)
Hispanic	<5 (<20%)
AAPI	0 (0%)
Other	<5 (<20%)
**Smoking Status**	
Never	18 (72%)
Current or Former	7 (28%)
**Obesity**	
No	12 (48%)
Yes	13 (52%)
**Comorbidities**[b]	
Cardiovascular	6 (24%)
Pulmonary	5 (20%)
Renal Disease	<5 (<20%)
Diabetes mellitus	11 (44%)
**ECOG Performance Status**	
0	5 (20%)
1	10 (40%)
2+	X*
Unknown	10 (40%)*
**Receptor status**	
HR+/HER2-	18 (72%)
HR+/HER2+	5 (20%)
HR+/HER2+	X*
Triple Negative	0 (0%)
Missing/unknown	2 (8%)*
**Cancer Status**	
Remission or NED, >5 years	<5 (<20%)
Remission or NED, <5 years	6 (24%)
Active and responding	<5 (<20%)
Active and stable	<5 (<20%)
Active and progressing	5 (20%)

medRxiv preprint doi: https://doi.org/10.1101/2023.03.09.23287038; this version posted March 10, 2023. The copyright holder for this preprint (which was not certified by peer review) is the author/funder, who has granted medRxiv a license to display the preprint in perpetuity.
It is made available under a CC-BY 4.0 International license .

Unknown	3 (12%)
**Timing of anti-cancer therapy**	
Never/After COVID-19	<5 (<20%)
0-4 weeks	17 (68%)
1-3 months	0 (0%)
>3 months	<5 (<20%)
Missing/unknown	1 (4%)
**Modality of active anti-cancer therapy** [b,c]	
None	7 (28%)
Chemotherapy	6 (24%)
Targeted Therapy	6 (24%)
Endocrine Therapy	10 (40%)
Immunotherapy	0 (0%)
Local (Surgery/Radiation)	<5 (<20%)
Other	0 (0%)
Missing/unknown	1 (4%)
**Region**	
Northeast	11 (44%)
Midwest	<5 (<20%)
South	<5 (<20%)
West	7 (28%)
Undesignated	0 (0%)
**Period of COVID-19 diagnosis**	
Jan-Apr 2020	10 (40%)
May-Aug 2020	9 (36%)
Sept-Dec 2020	5 (20%)
**Area of patient residence**	
Urban	9 (36%)
Suburban	8 (32%)
Rural	0 (0%)
Missing/unknown	8 (32%)
**Severity of COVID19**	
Mild	11 (44%)
Moderate/Severe	14 (56%)
***Cells combined to mask N<5 according to CCC19 low count policy**	
[a] Age was truncated at 90 years.	
[b] Percentages could sum to >100% because categories are not mutually exclusive.	
[c] Within 3 months of COVID-19 diagnosis.	
Variable Categories with one to five cases are masked by replacing with N < 5 according to CCC19 policy	

medRxiv preprint doi: https://doi.org/10.1101/2023.03.09.23287038; this version posted March 10, 2023. The copyright holder for this preprint (which was not certified by peer review) is the author/funder, who has granted medRxiv a license to display the preprint in perpetuity.
It is made available under a CC-BY 4.0 International license .

Suppl Table IVB: Unadjusted rates of outcomes after COVID-19 diagnosis among males with BC	
Outcomes	
Total all-cause mortality	5 (20)
30-day all-cause mortality	5 (20)
Received mechanical ventilation	<5 (<20%)
Admitted to an intensive care unit	<5 (<20%)
Admitted to the hospital	15 (60)
Clinical Complications	
Any cardiovascular complication [a]	<5 (<20%)
Any pulmonary complication [b]	12 (48)
Any gastrointestinal complication [c]	0 (0%)
Acute kidney injury	<5 (<20%)
Multisystem organ failure	<5 (<20%)
Superimposed infection	<5 (<20%)
Sepsis	<5 (<20%)
Any bleeding	<5 (<20%)
Interventions	
Remdesivir	<5 (<20%)
Hydroxychloroquine	7 (28)
Corticosteroids	<5 (<20%)
Other	9 (36)
Supplemental oxygen	12 (48)

Variable Categories with one to five cases are masked by replacing with N < 5 according to CCC19 policy

[a] Cardiovascular complication includes hypotension, myocardial infarction, other cardiac ischemia, atrial fibrillation, ventricular fibrillation, other cardiac arrhythmia, cardiomyopathy, congestive heart failure, pulmonary embolism (PE), deep vein thrombosis (DVT), stroke, thrombosis NOS complication.

[b] Pulmonary complication includes respiratory failure, pneumonitis, pneumonia, acute respiratory distress syndrome (ARDS), PE, pleural effusion, empyema.

[c] Gastrointestinal complication includes acute hepatic injury, ascites, bowel obstruction, bowel perforation, ileus, peritonitis

971

medRxiv preprint doi: https://doi.org/10.1101/2023.03.09.23287038; this version posted March 10, 2023. The copyright holder for this preprint (which was not certified by peer review) is the author/funder, who has granted medRxiv a license to display the preprint in perpetuity.
It is made available under a CC-BY 4.0 International license .

APPENDIX I:

List of Participants by Institution

Alphabetical list of participants by institution that contributed at least one record to the analysis.

Bolded = site PI/co-PIs; site co-investigators are listed alphabetically by last name.

- **Balazs Halmos, MD; Amit Verma, MBBS**; Benjamin A. Gartrell, MD; Sanjay Goel, MBBS; Nitin Ohri, MD; R. Alejandro Sica, MD; Astha Thakkar, MD (Albert Einstein College of Medicine, Montefiore Medical Center, Bronx, NY, USA)
- **Keith E. Stockerl-Goldstein, MD**; Omar Butt, MD, PhD; Jian Li Campian, MD, PhD; Mark A. Fiala, MSW; Jeffrey P. Henderson, MD, PhD; Ryan S. Monahan, MBA; Alice Y. Zhou, MD, PhD (Alvin J. Siteman Cancer Center at Washington University School of Medicine and Barnes-Jewish Hospital, St. Louis, MO, USA)
- **Michael A. Thompson, MD, PhD, FASCO**; Pamela Bohachek, RN, CCRC; Daniel Mundt, MD; Mitrianna Streckfuss, MPH; Eyob Tadesse, MD (Aurora Cancer Care, Advocate Aurora Health, Milwaukee, WI, USA)
- **Philip E. Lammers, MD, MSCI** (Baptist Cancer Center, Memphis, TN, USA)
- **Sanjay G. Revankar, MD, FIDSA** (The Barbara Ann Karmanos Cancer Institute at Wayne State University School of Medicine, Detroit, MI, USA)
- **Jaymin M. Patel, MD**; Andrew J. Piper-Vallillo, MD; Poorva Bindal, MBBS (Beth Israel Deaconess Medical Center, Boston, MA, USA)
- **Orestis A. Panagiotou, MD, PhD**; Pamela C. Egan, MD; Dimitrios Farmakiotis, MD, FACP, FIDSA; Hina Khan, MD; Adam J. Olszewski, MD (Brown University and Lifespan Cancer Institute, Providence, RI, USA)
- **Arturo Loaiza-Bonilla, MD, MSEd, FACP** (Cancer Treatment Centers of America, AZ/GA/IL/OK/PA, USA)
- **Salvatore A. Del Prete, MD**; Michael H. Bar, MD, FACP; Anthony P. Gulati, MD; K. M. Steve Lo, MD; Suzanne J. Rose, MS, PhD, CCRC, FACRP; Jamie Stratton, MD; Paul L. Weinstein, MD (Carl & Dorothy Bennett Cancer Center at Stamford Hospital, Stamford, CT, USA)
- **Robin A. Buerki, MD**; Jorge A. Garcia, MD, FACP (Case Comprehensive Cancer Center at Case Western Reserve University/University Hospitals, Cleveland, OH, USA)
- **Shilpa Gupta, MD**; Nathan A. Pennell, MD, PhD, FASCO; Manmeet S. Ahluwalia, MD, FACP; Scott J. Dawsey, MD; Christopher A. Lemmon, MD; Amanda Nizam, MD (Cleveland Clinic, Cleveland, OH, USA)
- **Claire Hoppenot, MD; Ang Li, MD, MS** (Dan L Duncan Comprehensive Cancer Center at Baylor College of Medicine, Houston, TX, USA)
- **Toni K. Choueiri, MD**; Ziad Bakouny, MD, MSc; Jean M. Connors, MD; George D. Demetri, MD, FASCO; Dory A. Freeman, BS; Antonio Giordano, MD, PhD; Chris Labaki, MD; Alicia K. Morgans, MD, MPH; Anju Nohria, MD; Andrew L. Schmidt, MD; Eliezer M. Van Allen, MD; Pier Vitale Nuzzo, MD, PhD; Wenxin (Vincent) Xu, MD; Rebecca L. Zon, MD (Dana-Farber Cancer Institute, Boston, MA, USA) (Dana-Farber Cancer Institute, Boston, MA, USA)
- **Susan Halabi, PhD, FASCO**; Tian Zhang, MD, MHS (Duke Cancer Institute at Duke University Medical Center, Durham, NC, USA)
- **John C. Leighton Jr, MD, FACP** (Einstein Healthcare Network, Philadelphia, PA, USA)
- **Gary H. Lyman, MD, MPH, FASCO, FRCP**; Jerome J. Graber MD, MPH; Petros Grivas, MD, PhD; Elizabeth T. Loggers, MD, PhD; Ryan C. Lynch, MD; Elizabeth S. Nakasone, MD, PhD; Michael T. Schweizer, MD; Lisa Tachiki, MD; Shaveta Vinayak, MD, MS; Michael J. Wagner, MD; Albert Yeh, MD (Fred Hutchinson Cancer Research Center/University of Washington/Seattle Cancer Care Alliance, Seattle, WA, USA)
- **Sharad Goyal, MD; Minh-Phuong Huynh-Le, MD, MAS** (George Washington University, Washington, DC, USA)
- **Lori J. Rosenstein, MD** (Gundersen Health System, WI, USA)
- **Peter Paul Yu, MD, FACP, FASCO**; Jessica M. Clement, MD; Ahmad Daher, MD; Mark E. Dailey, MD; Rawad Elias, MD; Asha Jayaraj, MD; Emily Hsu, MD; Alvaro G. Menendez, MD; Oscar K. Serrano, MD, MBA, FACS (Hartford HealthCare Cancer Institute, Hartford, CT, USA)
- **Clara Hwang, MD**; Shirish M. Gadgeel, MD; Sunny R K Singh, MD (Henry Ford Cancer Institute, Henry Ford Hospital, Detroit, MI, USA)

medRxiv preprint doi: https://doi.org/10.1101/2023.03.09.23287038; this version posted March 10, 2023. The copyright holder for this preprint (which was not certified by peer review) is the author/funder, who has granted medRxiv a license to display the preprint in perpetuity.
It is made available under a CC-BY 4.0 International license .

- **Melissa K. Accordino, MD, MS**; Divaya Bhutani, MD; Jessica E. Hawley, MD; Dawn Hershman, MD, MS, FASCO; Gary K. Schwartz, MD (Herbert Irving Comprehensive Cancer Center at Columbia University, New York, NY, USA)
- **Daniel Y. Reuben, MD, MS**; Mariam Alexander, MD, PhD; Sara Matar, MD; Sarah Mushtaq, MD (Hollings Cancer Center at the Medical University of South Carolina, Charleston, SC, USA)
- **Eric H. Bernicker, MD** (Houston Methodist Cancer Center, Houston, TX, USA)
- **John F. Deeken, MD**; Danielle Shafer, DO (Inova Schar Cancer Institute, Fairfax, VA, USA)
- **Mark A. Lewis, MD; Terence D. Rhodes, MD, PhD**; David M. Gill, MD; Clarke A. Low, MD (Intermountain Health Care, Salt Lake City, UT, USA)
- **Sandeep H. Mashru, MD**; Abdul-Hai Mansoor, MD (Kaiser Permanente Northwest, OR/WA, USA)
- **Brandon Hayes-Lattin, MD, FACP**; Aaron M. Cohen, MD, MS; Shannon McWeeney, PhD; Eneida R. Nemecek, MD, MS, MBA; Staci P. Williamson, BS (Knight Cancer Institute at Oregon Health and Science University, Portland, OR, USA)
- **Howard A. Zaren, MD, FACS**; Stephanie J. Smith, RN, MSN, OCN (Lewis Cancer & Research Pavilion @ St. Joseph's/Candler, Savannah, GA, USA)
- **Gayathri Nagaraj, MD**; Mojtaba Akhtari, MD; Eric Lau, DO; Mark E. Reeves, MD, PhD (Loma Linda University Cancer Center, Loma Linda, CA, USA)
- **Stephanie Berg, DO**; Natalie Knox (Loyola University Medical Center, Maywood, IL, USA)
- **Firas H. Wehbe, MD, PhD**; Jessica Altman, MD; Michael Gurley, BA; Mary F. Mulcahy, MD (Lurie Cancer Center at Northwestern University, Chicago, IL, USA)
- **Eric B. Durbin, DrPH, MS** (Markey Cancer Center at the University of Kentucky, Lexington, KY, USA)
- **Amit A. Kulkarni, MD**; Heather H. Nelson, PhD, MPH; Zohar Sachs, MD, PhD (Masonic Cancer Center at the University of Minnesota, Minneapolis, MN, USA)
- **Rachel P. Rosovsky, MD, MPH; Kerry L. Reynolds, MD**; Aditya Bardia, MD; Genevieve Boland, MD, PhD, FACS; Justin F. Gainor, MD; Leyre Zubiri, MD, PhD (Massachusetts General Hospital Cancer Center, Boston, MA, USA)
- **Thorvardur R. Halfdanarson, MD**; Tanios S. Bekaii-Saab, MD, FACP; Aakash Desai, MD, MPH; Surbhi Shah, MD; Zhuoer Xie, MD, MS (Mayo Clinic, AZ/FL/MN, USA) (Mayo Clinic, AZ/FL/MN, USA)
- **Ruben A. Mesa, MD, FACP**; Mark Bonnen, MD; Daruka Mahadevan, MD, PhD; Amelie G. Ramirez, DrPH, MPH; Mary Salazar, DNP, MSN, RN, ANP-BC; Dimpy P. Shah, MD, PhD; Pankil K. Shah, MD, MSPH (Mays Cancer Center at UT Health San Antonio MD Anderson Cancer Center, San Antonio, TX, USA)
- **Gregory J. Riely, MD, PhD; Elizabeth V. Robilotti MD, MPH**; Rimma Belenkaya, MA, MS; John Philip, MS (Memorial Sloan Kettering Cancer Center, New York, NY, USA)
- **Bryan Faller, MD** (Missouri Baptist Medical Center, St. Louis, MO, USA)
- **Rana R. McKay, MD**; Archana Ajmera, MSN, ANP-BC, AOCNP; Sharon S. Brouha, MD, MPH; Angelo Cabal, BS; Sharon Choi, MD, PhD; Albert Hsiao, MD, PhD; Jun Yang Jiang, MD; Seth Kligerman, MD; Taylor K. Nonato; Erin G. Reid, MD (Moores Comprehensive Cancer Center at the University of California, San Diego, La Jolla, CA, USA)
- **Lisa B. Weissmann, MD**; Chinmay Jani, MD; Carey C. Thomson, MD, FCCP, MPH (Mount Auburn Hospital, Cambridge, MA, USA)
- **Jeanna Knoble, MD**; Mary Grace Glace, RN; Cameron Rink, PhD, MBA; Karen Stauffer, RN; Rosemary Zacks, RN (Mount Carmel Health System, Columbus, OH, USA)
- **Sibel Blau, MD** (Northwest Medical Specialties, Tacoma, WA, USA)
- **Daniel G. Stover, MD**; Daniel Addison, MD; James L. Chen, MD; Margaret E. Gatti-Mays, MD; Sachin R. Jhawar, MD; Vidhya Karivedu, MBBS; Joshua D. Palmer, MD; Sarah Wall, MD; Nicole O. Williams, MD (The Ohio State University Comprehensive Cancer Center, Columbus, OH, USA)
- **Monika Joshi, MD, MRCP**; Hyma V. Polimera, MD; Lauren D. Pomerantz; Marc A. Rovito, MD, FACP (Penn State Health/Penn State Cancer Institute/St. Joseph Cancer Center, PA, USA)
- **Elizabeth A. Griffiths, MD**; Amro Elshoury, MBBCh (Roswell Park Comprehensive Cancer Center, Buffalo, NY, USA)
- **Salma K. Jabbour, MD**; Christian F. Misdary, MD; Mansi R. Shah, MD (Rutgers Cancer Institute of New Jersey at Rutgers Biomedical and Health Sciences, New Brunswick, NJ, USA)
- **Babar Bashir, MD, MS**; Christopher McNair, PhD; Sana Z. Mahmood, BA, BS; Vasil Mico, BS; Andrea Verghese Rivera, MD (Sidney Kimmel Cancer Center at Thomas Jefferson University, Philadelphia, PA, USA)

medRxiv preprint doi: https://doi.org/10.1101/2023.03.09.23287038; this version posted March 10, 2023. The copyright holder for this preprint (which was not certified by peer review) is the author/funder, who has granted medRxiv a license to display the preprint in perpetuity.
It is made available under a CC-BY 4.0 International license .

- **Sumit A. Shah, MD, MPH**; Elwyn C. Cabebe, MD; Michael J. Glover, MD; Alokkumar Jha, PhD; Ali Raza Khaki, MD; Lidia Schapira, MD, FASCO; Julie Tsu-Yu Wu, MD, PhD (Stanford Cancer Institute at Stanford University, Palo Alto, CA, USA)
- **Suki Subbiah, MD** (Stanley S. Scott Cancer Center at LSU Health Sciences Center, New Orleans, LA, USA)
- **Daniel B. Flora, MD, PharmD**; Goetz Kloecker, MD; Barbara B. Logan, MS; Chaitanya Mandapakala, MD (St. Elizabeth Healthcare, Edgewood, KY, USA)
- **Gilberto de Lima Lopes Jr., MD, MBA, FAMS, FASCO** (Sylvester Comprehensive Cancer Center at the University of Miami Miller School of Medicine, Miami, FL, USA)
- **Karen Russell, MD, FACP**; Brittany Stith, RN, BSN, OCN, CCRP (Tallahassee Memorial Healthcare, Tallahassee, FL, USA)
- **Natasha C. Edwin, MD**; Melissa Smits, APC (ThedaCare Cancer Care, Appleton, WI, USA)
- **David D. Chism, MD**; Susie Owenby, RN, CCRP (Thompson Cancer Survival Center, Knoxville, TN, USA)
- **Deborah B. Doroshow, MD, PhD**; Matthew D. Galsky, MD; Michael Wotman, MD (Tisch Cancer Institute at the Icahn School of Medicine at Mount Sinai, New York, NY, USA)
- **Julie C. Fu, MD**; Alyson Fazio, APRN-BC; Kathryn E. Huber, MD; Mark H. Sueyoshi, MD (Tufts Medical Center Cancer Center, Boston and Stoneham, MA, USA)
- **Jonathan Riess, MD, MS**; Kanishka G. Patel, MD (UC Davis Comprehensive Cancer Center at the University of California at Davis, CA, USA)
- **Vadim S. Koshkin, MD**; Hala T. Borno, MD; Daniel H. Kwon, MD; Eric J. Small, MD; Sylvia Zhang, MS (UCSF Helen Diller Family Comprehensive Cancer Center at the University of California at San Francisco, CA, USA)
- **Samuel M. Rubinstein, MD; William A. Wood, MD, MPH**; Christopher Jensen, MD (UNC Lineberger Comprehensive Cancer Center, Chapel Hill, NC, USA)
- **Trisha M. Wise-Draper, MD, PhD**; Syed A. Ahmad, MD, FACS; Punita Grover, MD; Shuchi Gulati, MD; Jordan Kharofa, MD; Tahir Latif, MBBS, MBA; Michelle Marcum, MS; Hira G. Shaikh; MD (University of Cincinnati Cancer Center, Cincinnati, OH, USA)
- **Daniel W. Bowles, MD**; Christoper L. Geiger, MD (University of Colorado Cancer Center, Aurora, CO, USA)
- **Merry-Jennifer Markham, MD, FACP, FASCO**; Atlantis D. Russ, MD, PhD; Haneen Saker, MD (University of Florida Health Cancer Center, Gainesville, FL, USA)
- **Jared D. Acoba, MD**; Young Soo Rho, MD, CM (University of Hawai'i Cancer Center, Honolulu, HI, USA)
- **Lawrence E. Feldman, MD; Kent F. Hoskins, MD**; Gerald Gantt Jr., MD; Li C. Liu, PhD; Mahir Khan, MD; Ryan H. Nguyen, DO; Mary Pasquinelli, APN, DNP; Candice Schwartz, MD; Neeta K. Venepalli, MD, MBA (University of Illinois Hospital & Health Sciences System, Chicago, IL, USA)
- **Praveen Vikas, MD** (University of Iowa Holden Comprehensive Cancer Center, Iowa City, IA, USA)
- **Elizabeth Wulff-Burchfield, MD**; Anup Kasi MD, MPH (The University of Kansas Cancer Center, Kansas City, KS, USA)
- **Christopher R. Friese, PhD, RN, AOCN, FAAN; Leslie A. Fecher, MD** (University of Michigan Rogel Cancer Center, Ann Arbor, MI, USA)
- **Blanche H. Mavromatis, MD**; Ragneel R. Bijjula, MD; Qamar U. Zaman, MD (UPMC Western Maryland, Cumberland, MD, USA)
- **Jeremy L. Warner, MD, MS, FAMIA, FASCO**; Alaina J. Brown, MD, MPH; Alicia Beeghly-Fadiel, PhD; Alex Cheng, PhD; Sarah Croessmann, PhD; Elizabeth J. Davis, MD; Stephany N. Duda, PhD, MS; Kyle T. Enriquez, MSc BS; Benjamin French, PhD; Erin A. Gillaspie, MD, MPH; Daniel Hausrath, MD; Cassandra Hennessy, MS; Chih-Yuan Hsu, PhD; Douglas B. Johnson, MD, MSCI; Xuanyi Li, BA; Sanjay Mishra, MS, PhD; Sonya A. Reid, MD, MPH; Brian I. Rini, MD, FACP, FASCO; Yu Shyr, PhD; David A. Slosky, MD; Carmen C. Solorzano, MD, FACS; Tianyi Sun, MS; Matthew D. Tucker, MD; Karen Vega-Luna, MA; Lucy L. Wang, BA (Vanderbilt-Ingram Cancer Center at Vanderbilt University Medical Center, Nashville, TN, USA)
- **David M. Aboulafia, MD**; Brett A. Schroeder, MD (Virginia Mason Cancer Institute, Seattle, WA, USA)
- **Matthew Puc, MD**; Theresa M. Carducci, MSN, RN, CCRP; Karen J. Goldsmith, BSN, RN; Susan Van Loon, RN, CTR, CCRP (Virtua Health, Marlton, NJ, USA)
- **Umit Topaloglu, PhD, FAMIA**; Saif I. Alimohamed, MD (Wake Forest Baptist Comprehensive Cancer Center, Winston-Salem, NC, USA)
- **Robert L. Rice, MD, PhD** (WellSpan Health, York, PA, USA)
- **Prakash Peddi, MD; Lane R. Rosen, MD**; Briana Barrow McCollough, BSc, CCRC (Willis-Knighton Cancer Center, Shreveport, LA, USA)

medRxiv preprint doi: https://doi.org/10.1101/2023.03.09.23287038; this version posted March 10, 2023. The copyright holder for this preprint (which was not certified by peer review) is the author/funder, who has granted medRxiv a license to display the preprint in perpetuity.
It is made available under a CC-BY 4.0 International license .

- **Mehmet A. Bilen, MD**; Cecilia A. Castellano; Deepak Ravindranathan, MD, MS (Winship Cancer Institute of Emory University, Atlanta, GA, USA)
- **Navid Hafez, MD, MPH**; Roy Herbst, MD, PhD; Patricia LoRusso, DO, PhD; Maryam B. Lustberg, MD, MPH; Tyler Masters, MS; Catherine Stratton, BA (Yale Cancer Center at Yale University School of Medicine, New Haven, CT, USA) (Yale Cancer Center at Yale University School of Medicine, New Haven, CT, USA)
